# Sequencing of Kaposi’s Sarcoma Herpesvirus (KSHV) genomes from persons of diverse ethnicities and provenances with KSHV-associated diseases demonstrate multiple infections, novel polymorphisms, and low intra-host variance

**DOI:** 10.1371/journal.ppat.1012338

**Published:** 2024-07-15

**Authors:** Vickie A. Marshall, Elena M. Cornejo Castro, Charles A. Goodman, Nazzarena Labo, Isabella Liu, Nicholas C. Fisher, Kyle N. Moore, Ananthakrishnan Nair, Taina Immonen, Brandon F. Keele, Mark N. Polizzotto, Thomas S. Uldrick, Yunxiang Mu, Tanuja Saswat, Laurie T. Krug, Kevin M. McBride, Kathryn Lurain, Ramya Ramaswami, Robert Yarchoan, Denise Whitby

**Affiliations:** 1 Viral Oncology Section, AIDS and Cancer Virus Program, Frederick National Laboratory for Cancer Research, Frederick, Maryland, United States of America; 2 Retroviral Evolution Section, AIDS and Cancer Virus Program, Frederick National Laboratory for Cancer Research, Frederick, Maryland, United States of America; 3 HIV and AIDS Malignancy Branch, National Cancer Institute, Bethesda, Maryland, United States of America; 4 Department of Epigenetics and Molecular Carcinogenesis, The University of Texas MD Anderson Cancer Center, Houston, Texas, United States of America; Leibniz Institute of Virology (LIV), GERMANY

## Abstract

Recently published near full-length KSHV genomes from a Cameroon Kaposi sarcoma case-control study showed strong evidence of viral recombination and mixed infections, but no sequence variations associated with disease. Using the same methodology, an additional 102 KSHV genomes from 76 individuals with KSHV-associated diseases have been sequenced. Diagnoses comprise all KSHV-associated diseases (KAD): Kaposi sarcoma (KS), primary effusion lymphoma (PEL), KSHV-associated large cell lymphoma (KSHV-LCL), a type of multicentric Castleman disease (KSHV-MCD), and KSHV inflammatory cytokine syndrome (KICS). Participants originated from 22 different countries, providing the opportunity to obtain new near full-length sequences of a wide diversity of KSHV genomes. These include near full-length sequence of genomes with KSHV K1 subtypes A, B, C, and F as well as subtype E, for which no full sequence was previously available. High levels of recombination were observed. Fourteen individuals (18%) showed evidence of infection with multiple KSHV variants (from two to four unique genomes). Twenty-six comparisons of sequences, obtained from various sampling sites including PBMC, tissue biopsies, oral fluids, and effusions in the same participants, identified near complete genome conservation between different biological compartments. Polymorphisms were identified in coding and non-coding regions, including indels in the K3 and K15 genes and sequence inversions here reported for the first time. One such polymorphism in KSHV ORF46, specific to the KSHV K1 subtype E2, encoded a mutation in the leucine loop extension of the uracil DNA glycosylase that results in alteration of biochemical functions of this protein. This confirms that KSHV sequence variations can have functional consequences warranting further investigation. This study represents the largest and most diverse analysis of KSHV genome sequences to date among individuals with KAD and provides important new information on global KSHV genomics.

## Author summary

The prevalence of KSHV infection, one of eight viruses associated with human cancer, differs geographically as does the incidence of Kaposi sarcoma and other KSHV-associated diseases. Distribution of KSHV subtypes, defined solely by the sequence of the K1 gene, also differs by region. One hundred and two near full-length KSHV genomes from people with associated diseases from a wide variety of countries were sequenced, substantially increasing the number and diversity of KSHV genomes publicly available, thereby allowing the analysis of variance across geographical regions, potential contributions of viral genetics to the risk of disease, and viral evolution. Our data demonstrate that viral recombination is common, as previously published, and that infections with multiple KSHV genomes may also be frequent, which has not been widely reported. We did not find evidence of an association between viral subtypes and disease nor indications that different sampling sites from the same individual, including tumor specimens, harbored substantially different KSHV genomes. Our data significantly inform the current efforts to study epidemiology and the prevention of KSHV infection, including vaccine development, as well as the pathogenesis of associated diseases.

## Introduction

KSHV, also known as human herpesvirus 8, is the causative agent of Kaposi sarcoma (KS), primary effusion lymphoma (PEL), KSHV-associated large cell lymphoma, a type of multicentric Castleman disease (KSHV-MCD), and KSHV inflammatory cytokine syndrome (KICS) [1] collectively known as KSHV associated diseases (KAD). KSHV prevalence varies geographically and is often mirrored by the incidence of associated cancers [2].

KSHV is a large, double-stranded DNA virus that encodes 80+ proteins, microRNAs, and other non-coding RNAs all of which may contribute to oncogenesis. Historically, genetic analysis has been limited to specific variable KSHV genes, primarily K1 and K15, which constitute less than 2% of the KSHV genome, excluding the long terminal repeats. KSHV subtypes, as determined by sequence variations in the K1 gene, have distinct geographical distributions. Subtypes A and C are common in Europe and North America, whilst B and A5 variants are predominantly found in sub-Saharan Africa. KSHV D, E and F subtypes were first identified in isolated populations in the Pacific, in indigenous South American peoples, and among the San people of Botswana, respectively, but are starting to be reported more broadly [3–5]. KSHV K15 gene subtypes include the predominant P, found worldwide, as well as the less frequently identified M and N subtypes; the M subtype has been reported in Japan, the Americas, and Africa, whereas the N subtype appears to be more common in Africa based upon currently available sequence information [6–10].

Despite recent advances in next-generation sequencing (NGS) technologies increasing the number of complete and partial KSHV genomes available for study, complete genomes of the K1 D and E subtypes are still unavailable, likely due to their rare occurrence and distribution in geographical isolated populations [4,11]. As KSHV is endemic in sub-Saharan Africa and infected individuals frequently have higher viral loads facilitating sequencing efforts, the majority of KSHV genomes deposited are isolated from African individuals. In our recent Cameroon KS case-control study, where we observed predominantly B1 and A5 K1 subtypes in whole blood and oral fluids, we were unable to identify sequence variations associated with the risk of KS. However, we observed evidence of multiple infections in three individuals [12].

Recognizing the need to increase the number and diversity of KSHV genomes available for study, we have used our Agilent SureSelect^xt^ target enrichment protocol to sequence KSHV genomes from 78 individuals enrolled in the HIV and AIDS Malignancy Branch of the National Cancer Institute (NCI). These individuals were of diverse ethnicities, and all had at least one, and often multiple, KAD [13]. Study participants were born in 22 different countries on 5 continents. Nested within this study is an analysis of KSHV genomes obtained from different biological compartments of 26 individuals, looking for evidence of sequence variation across different sampling sites. A total of 102 KSHV genomes were obtained. To our knowledge, this is the largest and most comprehensive analysis of KSHV genome diversity to date. Thus, this study expands the current knowledge of the molecular diversity of KSHV genomes, allows the investigation of sequence variations in coding and non-coding regions and their potential clinical significance, and contributes to the understanding of the evolution of KSHV.

## Results

### Genome diversity observed within the study population

One hundred and two near full-length KSHV genomes were successfully obtained from 76 of 78 study participants, including longitudinal and paired-sample genome comparisons (S1 Table). Individuals in the study all had histories of KAD as described in Table 1. Most participants were men (87.5%) living with HIV (88%) and identified themselves as men who have sex with men (MSM). KS was the most common KSHV-associated disease, affecting 87% of the participants. Diagnoses at time of sample collection included KS (20.5%), PEL (2.6%), and MCD (5.1%) in isolation, but many participants had multiple diagnoses such as KS-PEL, KS-MCD, or KS-KICS (50%). Some individuals had combined diagnoses of KS-PEL-MCD or KS-PEL-KICS (11.5%). Sequences from two participants, FNL0017 and FNL0091, had insufficient read depth coverage (S2 Table) and were not included in downstream analyses.

**Table 1:** Participant Characteristics.

ID	Sex	Ethnicity	Geographical region of Birth	HIV Status	KAD History
FNL002	Male	White	North America	HIV pos	PEL
FNL003	Male	African American	North America	HIV pos	KS, PEL
FNL008	Male	African American	North America	HIV pos	KS, MCD
FNL010	Male	White	North America	HIV pos	KS, KICS
FNL013	Male	Hispanic/Latino	Central America	HIV pos	KS, PEL, KICS
FNL0015	Male	White	North America	HIV pos	MCD
FNL0016	Male	Hispanic/Latino	Central America	HIV pos	KS
FNL0017	Male	African American	North America	HIV pos	MCD
FNL0018	Male	African American	North America	HIV pos	KS, KICS
FNL0019	Male	White	North America	HIV pos	MCD
FNL0020	Male	White	North America	HIV pos	KS, MCD
FNL0021	Male	White	North America	HIV pos	KS, MCD
FNL0022	Male	White	North America	HIV pos	KS, KICS
FNL0023	Male	White	North America	HIV pos	MCD
FNL0024	Male	African American	North America	HIV neg	KS
FNL0025	Male	White	North America	HIV pos	KS, MCD
FNL0026	Male	African	Western Africa	HIV pos	PEL
FNL0027	Male	Latino	South America	HIV pos	KS, PEL
FNL0028	Female	African	Western Africa	HIV pos	KS, KICS
FNL0029	Male	African American	North America	HIV pos	KS
FNL0030	Female	African	Western Africa	HIV pos	KS, MCD
FNL0031	Male	White	Western Asia	HIV neg	KS
FNL0032	Male	White	North America	HIV pos	MCD
FNL0033	Male	African American	North America	HIV pos	KS
FNL0034	Male	African American	North America	HIV pos	KS, PEL, MCD
FNL0035	Female	African American	North America	HIV pos	KS, KICS
FNL0036	Male	African American	North America	HIV pos	KS, KICS
FNL0037	Male	White	North America	HIV pos	KS
FNL0038	Male	Hispanic/Latino	Caribbean	HIV pos	KS
FNL0039	Male	African	Eastern Africa	HIV pos	KSHV-LCL, MCD
FNL0040	Male	Hispanic/Latino	North America	HIV pos	KS, KICS
FNL0041	Male	White	North America	HIV pos	KS, KICS
FNL0042	Male	African American	North America	HIV pos	KS, PEL
FNL0043	Male	White	North America	HIV pos	KS, MCD
FNL0044	Male	White	North America	HIV pos	KS, PEL
FNL0045	Male	African American	North America	HIV pos	KS, KICS
FNL0046	Male	White	North America	HIV pos	KS
FNL0047	Female	African	Western Africa	HIV pos	KS, PEL, MCD
FNL0048	Male	African	Western Africa	HIV pos	KS, PEL, KICS
FNL0049	Male	White	Southern Europe	HIV neg	KS
FNL0050	Male	White	North America	HIV pos	KS, KICS
FNL0051	Male	White	North America	HIV neg	KS
FNL0052	Male	Hispanic/Latino	Central America	HIV pos	KS, KICS
FNL0053	Male	African American	North America	HIV pos	KS, KICS
FNL0054	Male	White	North America	HIV pos	KS
FNL0055	Male	White	North America	HIV pos	KS, MCD
FNL0056	Male	African American	North America	HIV pos	KS, PEL
FNL0059	Male	African American	North America	HIV pos	KS, KICS
FNL0060	Female	African	Eastern Africa	HIV pos	KS, KICS
FNL0061	Male	White	North America	HIV pos	KS, PEL, MCD
FNL0062	Male	Indian	Caribbean	HIV pos	KS, MCD
FNL0063	Female	African	Eastern Africa	HIV pos	KS, MCD
FNL0064	Female	African	Middle Africa	HIV pos	KS, KICS
FNL0065	Male	White	Southern Europe	HIV neg	KS
FNL0066	Male	Hispanic/Latino	Central America	HIV pos	KS, KICS
FNL0067	Male	Hispanic/Latino	Central America	HIV pos	KS, KICS
FNL0068	Male	Hispanic/Latino	South America	HIV neg	KS, MCD
FNL0069	Male	White	North America	HIV pos	KS, PEL, MCD
FNL0070	Male	Hispanic/Latino	Caribbean	HIV pos	KS
FNL0071	Male	White	Southern Europe	HIV neg	KS
FNL0072	Male	African American	North America	HIV neg	KS, KICS
FNL0073	Male	African American	North America	HIV pos	KS, PEL, MCD
FNL0074	Male	Hispanic/Latino	South America	HIV pos	KS, MCD
FNL0076	Male	Hispanic/Latino	Unknown	HIV pos	KS, KICS
FNL0077	Male	African American	North America	HIV pos	KS, MCD
FNL0078	Male	African American	North America	HIV pos	KS, PEL, MCD
FNL0079	Male	Asian	North America	HIV pos	KS
FNL0080	Male	Asian	Eastern Asia	HIV neg	KS
FNL0081	Male	African American	North America	HIV pos	KS, PEL
FNL0082	Male	African American	North America	HIV pos	KS, MCD
FNL0083	Male	Native American	North America	HIV pos	MCD
FNL0084	Male	White	North America	HIV pos	KS, PEL, MCD
FNL0085	Female	African	Western Africa	HIV pos	KS, MCD
FNL0086	Male	African American	North America	HIV pos	KS, MCD
FNL0088	Male	White	North America	HIV pos	KS, anal cancer
FNL0089	Male	African American	North America	HIV pos	KS, KICS
FNL0090	Male	African American	North America	HIV pos	MCD
FNL0091	Male	Multiracial	North America	HIV pos	KS

Characteristics of the study population. United Nations geographical designations were used to identify the region of birth. Current and past history of KAD up to the sample collection date is indicated. HIV = human immunodeficiency virus; KS = Kaposi sarcoma; MCD = multicentric Castleman disease; KICS = KSHV inflammatory cytokine syndrome; KSHV-LCL = KSHV associated large cell lymphoma.

**Fig 1:**
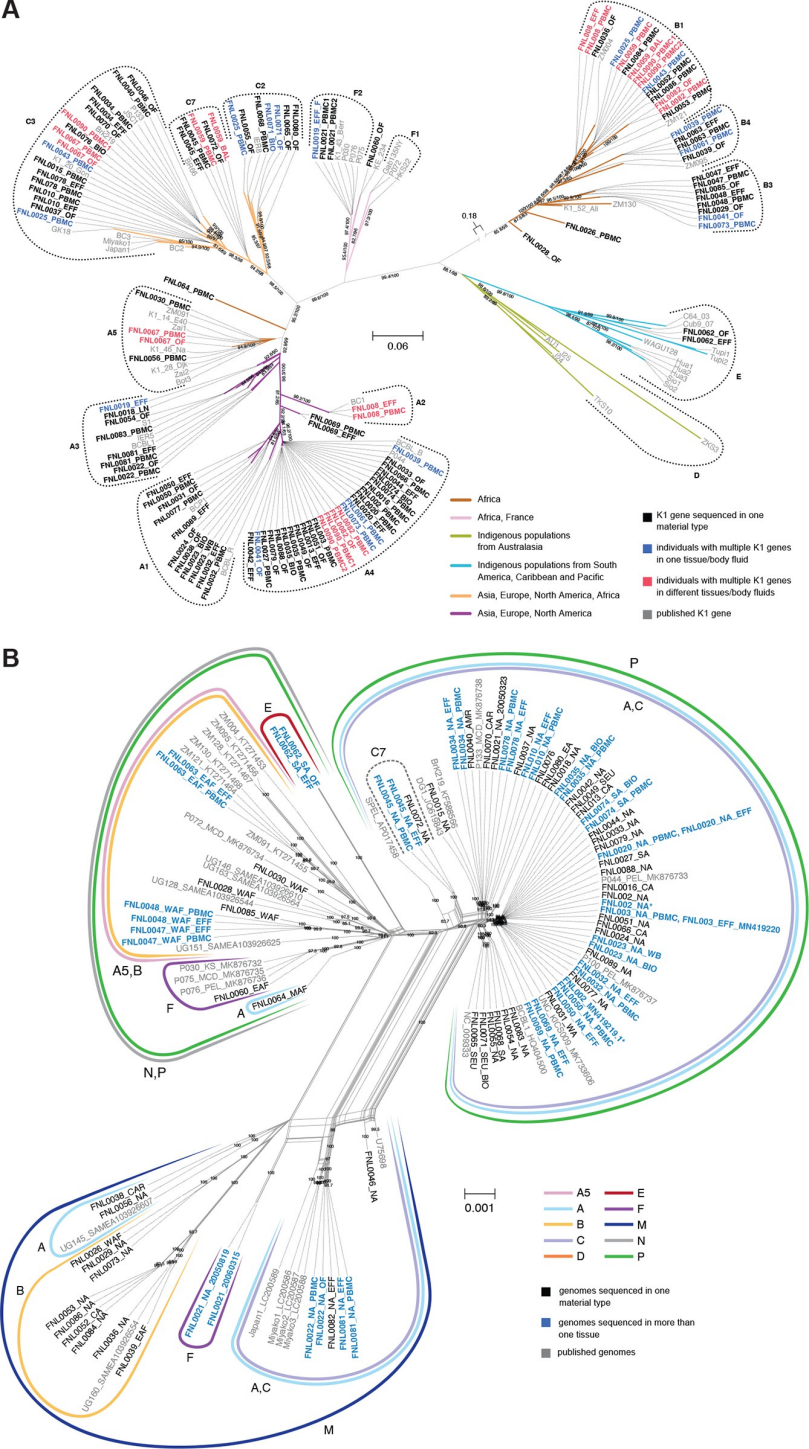
K1 Phylogenetic Analysis and SplitsTree Phylogenetic Analysis of KSHV Whole Genomes. **(A)** KSHV subtypes determined by the K1 gene amino acid (aa) sequence performed using IQTree with 1000 bootstrap replicates [44]. The analysis included 120 K1 amino acid sequences (344 aa) from current study individuals including multiple infections that were generated using *de novo* K1 sub-assembly. 52 published KSHV K1 aa sequences representing all subtypes A-F define the branches as indicated (shaded in grey). Current study samples were KSHV K1 subtypes A, B, C, E, and F. Study sequences are labeled as FNL and bolded in black, those samples with multiple infections are colored in blue, and multiple infections identified in more than one tissue from the same individual are red. **(B)** Near-full genome splits network for 85 current study KSHV and 32 published genomes. A total of approximately 130,000 nucleotide positions were included in the final data set excluding 5 repetitive regions. Analysis included KSHV genomes assembled from longitudinal PBMC and multiple tissue comparisons. The KSHV genomes of 17 of 18 comparisons determined to not have significant sequence variations, highlighted in blue. FNL002, shown in red, was published in 2020 as a K1 A2 subtype. The subtype of the PBMC genome is consistent with K1 A4 as shown in Fig 1A. Bootstrap values >80 are as indicated. A phi test for recombination performed using SplitsTree indicated strong evidence of viral recombination (P-value < 0.001). Published genomes used for phylogenetic analysis: GK18 (NC_009333.1), BC-1 (U75698.1), Japan/Miyako sequences (LC200587.1-LC200589.1), BCBL-1 (HQ404500.1), JSC-1 (MK143395.1), BrK.219 (KF588566.1), Zambian sequences ZM007-ZM130 (KT271453-KT271468), P044 (MK876733.1), P100 (MK876737.1), P133 (MK876738.1), P030 (MK876732.1), P075 (MK876735.1), P076 (MK876736.1), P072 (MK876734.1), UNC_KICS009 (MK733606.1), ZM004 (KT271453), ZM091 (KT271455.1), ZM095 (KT271456.1), ZM128 (KT271467.1), ZM130 (KT271468.1), ZM121 (KT271464), UG130 (SAMEA103926549), UG145 (SAMEA103926607), UG151 (SAMEA103926625), UG160 (SAMEA103926554), UG128 (SAMEA103926544), UG146 (SAMEA103926610), UG163 (SAMEA103926564), DG1 (JQ619843.1), SPEL (AP017458), and BC-2 (AF133042.1).

We observed KSHV genomes with a wide variety of KSHV K1 gene subtypes as shown in Fig 1A and report the first near full-length sequences from genomes with K1 C7 and E2 subtypes. KSHV K1 subtypes were consistent with the regions of provenance of the study participants including North, Central, and South America, the Caribbean, Europe, Asia, and Africa. The phylogenetic analysis for two KSHV K1 gene sequences from western Africa (WAF) that fall within the B subtype, FNL0026_WAF and FNL0028_WAF, have distinctive polymorphisms that caused them to cluster separately. They were provided by individuals born in African countries from which limited or no KSHV sequence information has been published. Similarly, K1 gene sequences from middle Africa (MAF) and North America (NA), FNL0064_MAF and FNL0069_NA, could not be precisely classified within the A branch. Interestingly, FNL0060_EAF from eastern Africa clustered with a distinctive sample published from Kenya within the F subtype (Fig 1A). The 66 new KSHV genomes obtained in this study are available online in GenBank as accession numbers OR829339-OR829404.

Of the 102 near full-length KSHV genomes, sample consensuses from 10 individuals had to be excluded from analyses due to unresolvable mixed KSHV infections. Eighty-five genomes, including 18 generated as part of a multiple tissue comparison, and 30 other published genomes available as of April 2022 were included in a SplitsTree analysis (Fig 1B). The SplitsTree analysis shows conflicting phylogenetic signals, shown as a complex array of splits, with a PHI test supporting a high degree of viral recombination (P-value < 0.001) [14]. The most variable gene regions, K1 and K15, drive the phylogenic splits with the best supported split observed between samples with the K15 M and P, N alleles. A distance table created with near-full genomes indicated that the greatest variation in the current study was observed between genomes FNL0062_SA and FNL0036_NA (97.478 percent identity between K1 subtype E2 and B1 respectively); several independent samples share greater than 99% identity within the current study (e.g. K1 subtype A4 samples FNL0016_CA and FNL002_NA) as well as with publicly available viral genomes of the same or closely related subtypes (e.g. genomes FNL002_NA and P044_PEL) [5]. In fact, three publicly available KSHV genomes from Miyako share greater than 99.85% identity with each other (S4 Table) [7].

### Analysis of KSHV variations and disease type

A total of 1989 single nucleotide variations across 1788 positions were identified in 45 unique KSHV genomes using NC_009333.1 as the reference genome. The frequency of sequence variations occurring in genomes of certain K1 subtypes are visualized in S1 Fig. Variations occur across the viral genome and the patterns are similar across minor K1 subtypes (for instance between C1-C3). However, the pattern of polymorphisms does differ between major K1 subtypes (for instance between the B and C). Changes in the same genome positions can be noted in different subtypes, but those changes are often not identical.

**Fig 2:**
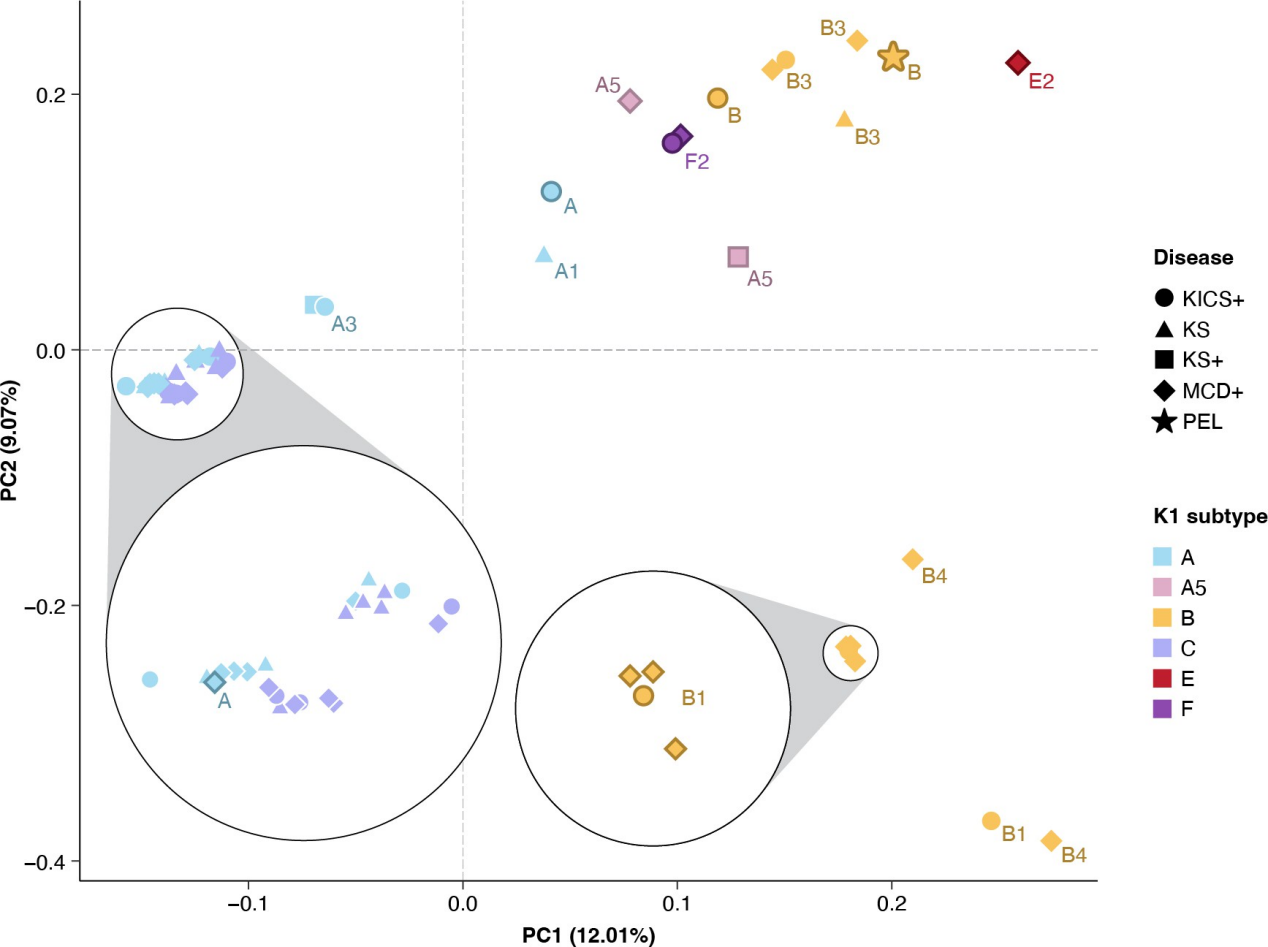
Principal Component Analysis (PCA) of sequence variations and KSHV-associated disease. Analysis included 1989 informative variants across 1788 positions that passed QC generated from a consensus alignment of 45 non-replicative genomes. KSHV K1 subtypes are colorized and associated diseases are indicated by symbols. No KSHV-associated diseases grouped together. Instead, the genomes segregated by subtype with associated diseases interspersed within each quadrant. Individual samples discussed in detail are highlighted by borders for emphasis.

A principal component analysis (PCA) was performed to assess whether the sequence variations observed within these genomes clustered specifically with either disease type or K1 subtype (Fig 2). PCA was performed using 45 unique KSHV genomes, and participant-level variables. Approximately 20% of the variance observed within the dataset can be explained by the first two principal components. No sequence-specific patterns were observed by disease type; genomes with similar K1 subtypes tend to be clustered together. The PCA does not precisely follow the topology shown in the phylogenetic analysis (Fig 1A) which only includes variations within the K1 gene. Notably, the genomes with K1 A and C subtype, known to cluster closely together in phylogenetic analyses, form a dense group in the PCA. Greater variance is observed in samples with K1 A5 and B subtypes. Five B1 subtypes, highlighted in the bottom right quadrant, have variations spanning the viral genome that cause them to cluster together with B4 subtypes but separately from other B genomes. Sequences of unclassified B subtypes from African countries with limited KSHV sequence information, FNL0026_WAF and FNL0028_WAF (western Africa), as well as the E2 FNL0062_SA (South America) and unclassified F subtype FNL0060_EAF (eastern Africa), cluster together in the upper right quadrant. While the unclassified A subtype genome, FNL0064_MAF (middle Africa) also segregates separately within the upper right-hand quadrant, FNL0069_NA (North America) shown in the lower left panel of the PCA analysis clusters near the A1-A4 subtype genomes.

### Tissue comparison analysis

For 26 participants, specimens from multiple tissues or compartments were available, allowing comparative analysis of near-full length viral genome sequences. For FNL002_NA and FNL003_NA, the newly generated KSHV genome sequences were compared to previously published genomes obtained from effusions (GenBank accessions MN419219.1 and MN419220.1)[15]. Samples from seven participants showed multiple infections and were dropped from analysis. An additional comparison was dropped due to one sample failing to have read depth coverage sufficient to generate a consensus sequence (FNL0041_NA, S2 Table). In seventeen out of eighteen remaining comparisons KSHV sequences collected from different tissues in each participant were nearly identical, with percent identities averaging 99.987%, and ranging between 99.91% and 100% (Fig 1B and S1 Table). Manual inspection of the sequences indicated that areas near repetitive regions and differences in read depth coverage contributed to the minor differences observed. This pattern of sequence conservation in the seventeen comparisons is also illustrated through K1 subtype specific variations as illustrated in S2 Fig.

However, for participant FNL002, it was determined that the KSHV genome obtained from a PEL effusion in March 2013 was of a different K1 subtype than the one obtained from a surveillance PBMC sample taken seven months later, consistent with a multiple infection, like the observation made in FNL0021_NA [15]. Although the genomes were 99.925% similar, they differed by 101 positions across the viral genome, particularly within the K1 gene region (S2 Fig). Further, the effusion genome encoded polymorphisms within the mature miR-K12-9 previously shown to adversely affect processing [16]. KSHV genomes can be near identical within closely related subtypes, as shown in S4 Table, and additional longitudinal sequencing from this individual would be required to support a multiple infection conclusion.

### Infection with multiple distinct KSHV genomes

**Fig 3:**
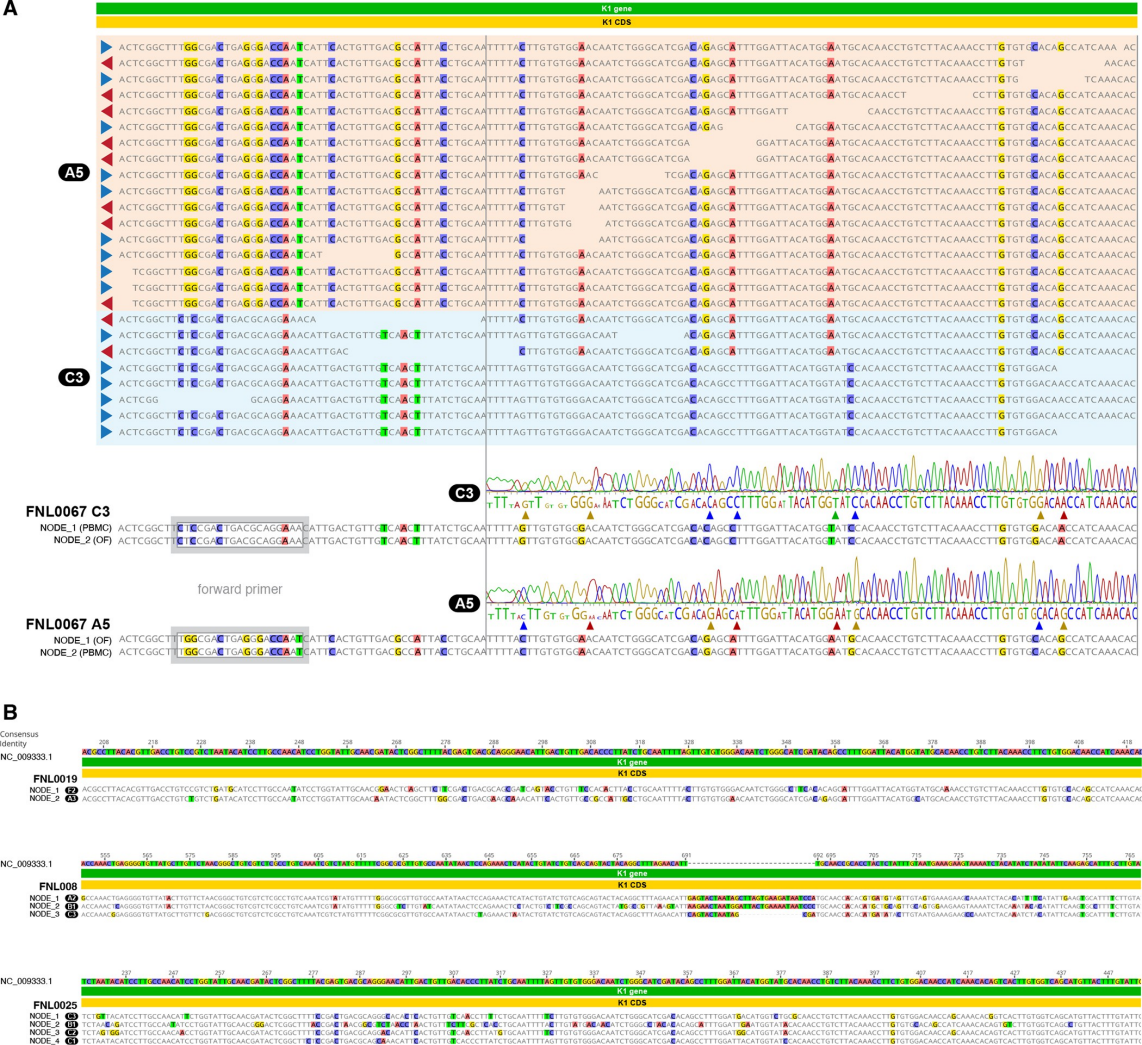
(**A)** Example of Sanger Sequence Confirmation of KSHV Multiple Infection: Screen shot of reference-guided assembly of reads within the KSHV K1 gene region for FNL0067 which is a multiple infection of an A5 and C3 genome as indicated shaded in peach and blue. K1 gene subtype specific nodes generated by de novo are shown for the C3 and A5 subtypes in both the oral fluid and PBMC material used for NGS. Sanger sequence subtype-specific traces used for confirmation for each subtype are aligned above. The position of the forward primer is boxed upstream of the beginning of the trace data (S3 Table). **(B)** Examples of *De Novo* Subassemblies of KSHV Multiple Infections: Screen shots of KSHV K1 gene alignments made in Geneious 2022.0.2 for three study samples with 2, 3, and 4 multiple infections indicated by de novo subassembly of K1 region-specific reads. The subtypes are indicated in black circles and variations indicated are highlighted in color determined by comparison to the NC_009333.1 reference genome, GK18.

Individual samples obtained from 14 participants were determined to harbor more than one distinct KSHV genome. Multiple infections were first identified through manual evaluation of reference-guided alignment of reads against NC_009333.1, with particular attention to the variable gene K1. Sanger sequencing using primers specific to distinct K1 gene subtypes (S3 Table) confirmed multiple infections in 11 of 14 participants as indicated in Table 2 and illustrated for participant FNL0067 in Fig 3A. Multiple infections were further validated through sequencing of additional samples available from seven participants, including longitudinally collected PBMCS (FNL0021 and FNL0090) and various other tissues (FNL008, FNL0039, FNL0059, FNL0067, FNL0082) (Fig 1A). For FNL0039_EAF and FNL0071_SEU, the frequency of the minor variant was low enough that a consensus could be successfully resolved for the major variant (Fig 1B). For 10 of 11 Sanger sequencing confirmed multiple-infected samples, contiguous major variant genomes could not be adequately resolved with reference-guided assembly (Table 2).

**Table 2:** Characteristics of Mixed Infections.

Sample	Sample/Date	KSHV Subtype 1 Major	KSHV Subtype 2 Minor	KSHV Subtype 3 Minor	KSHV Subtype 4 Minor	*Estimated % Frequency of Major/Minor genomes	Mean Coverage	KAD
FNL008_NA	Effusion / 2-6-13	**A2**	**B1**	C3		96.22 / 3.75	1088.26	KS, MCD
FNL008_NA	PBMC / 12-12-12	**A2**	B1			98.34 / 1.65	489.92	KS, MCD
FNL0019_NA	Effusion / 6-5-13	**F2**	**A3**			91.5 / 8.5	195.69	MCD
FNL0021_NA	PBMC/ 8-19-05	**F2**	**C3**			80.6 / 17.6	122.44	KS, MCD
FNL0021_NA	PBMC/ 3-15-06	**F2**	**C3**			57.8 / 40.1	6699.21	KS, MCD
FNL0025_NA	PBMC / 7-24-13	**C1**	**C2**	B1	C3	85.49 / 14.09	393.14	KS, MCD
FNL0039_EAF	PBMC / 10-26-20	**B4**	A4	C3		99.62 / 0.358	3256.2	KSHV-LCL, MCD
FNL0041_NA	Oral fluid / 11-28-18	**B3**	**A4**			98.8 / 0.98	8473.2	KS, KICS
FNL0043_NA	PBMC / 12-13-17	**C3**	**B1**			83.38 / 16.53	852.33	KS, MCD
FNL0059_NA	BAL / 7-30-20	**C7**	**B1**			83.66 / 16.23	114.5	KS, KICS
FNL0059_NA	PBMC / 8-10-20	**C7**	**B1**			84.41 / 15.42	340.25	KS, KICS
FNL0061_NA	PBMC / 4-28-21	**A4**	B4			94.53 / 5.45	972.37	KS, PEL, MCD
FNL0067_CA	Oral fluid / 4-10-19	**A5**	**C3**			79.57 / 20.4	37.96	KS, KICS
FNL0067_CA	PBMC / 9-4-19	**A5**	**C3**			93.18 / 6.8	155.79	KS, KICS
FNL0071_SEU	Oral fluid / 3-28-12	**C2**	**C1**			78.64 / 20.92	1385.75	KS
FNL0073_NA	PBMC / 2-14-18	**B3**	A4			98.15 / 1.7	787.71	KS, PEL, MCD
FNL0082_NA	Oral fluid / 6-23-21	**A4**	**B1**			57.65 / 37.74	1029.58	KS, MCD
FNL0082_NA	PBMC / 7-1-20	**A4**	**B1**			75.37 / 21.16	1212.78	KS, MCD
FNL0090_NA	PBMC / 4-1-19	**A4**	C3	**B1**		86.59 / 13.07	1809.24	MCD
FNL0090_NA	PBMC / 4-24-19	**A4**	**B1**			96.54 / 3.42	1240.1	MCD

Subtype determinations independently confirmed by Sanger sequencing using K1 gene-specific primers are shaded in gray. Sanger sequencing was successful in confirming 11 mixed infections either using original DNA or available alternative sample from the same individual. The estimated frequency of each infection is determined by the average frequency of nucleotides across all variant positions. For samples with more than two KSHV genomes, the minor variant frequency reported is a combination of all minor subtype reads. *The coverage was calculated on non-variant site identified with a p-value <0.00000024. In a 2018 KS biopsy from FNL0071, K1 subtype was classified as C1 [17] while in a 2012 PBMC sample from FNL0021, K1 subtype was classified as F2 [18] by Sanger sequencing. PBMC = peripheral blood mononuclear cells; KS = Kaposi sarcoma; BAL = bronchoalveolar lavage; PEL = primary effusion lymphoma; MCD = multicentric Castleman disease; KSHV-LCL = KSHV associated large cell lymphoma; KICS = KSHV inflammatory cytokine syndrome.

K1 and K15 gene subassemblies from the *de novo* assembly pipeline (S1 Document) later provided contiguous assemblies of these regions from samples with multiple infection (Fig 3B and S1 Table). Additionally, in participants with known multiple infections, broader patterns of within-host diversity across the near-full length KSHV genome were identified and validated by self-mapping sequencing reads against that sample’s consensus *de novo* assembly (S1 Document and S3A–S3D Fig). The nucleotide frequencies were plotted for all variant sites identified (640 positions [Interquartile range (IQR) = 256–1078]). Distinct and consistent patterns of nucleotide frequencies, corresponding to the major and minor genome variants contained within the sample, could be observed across the genome in all participants. A median variant frequency of 13% (IQR = 4%–20%) and 87% (IQR = 80%–96%) was detected for the minor and major genome variants, respectively. Among longitudinal samples, a shift in the minor genome variant frequency could be detected. For example, in FNL0090 the minor genome variant frequency decreases from 13% to 3% within 23 days during which time the individual was receiving treatment for their KAD (S3A Fig). Similar patterns of variant frequencies were detected between datasets obtained from different tissues sampled from the same participant (S3B Fig).

### Novel Features of KSHV Genomes associated with Geographic Variations

**Fig 4:**
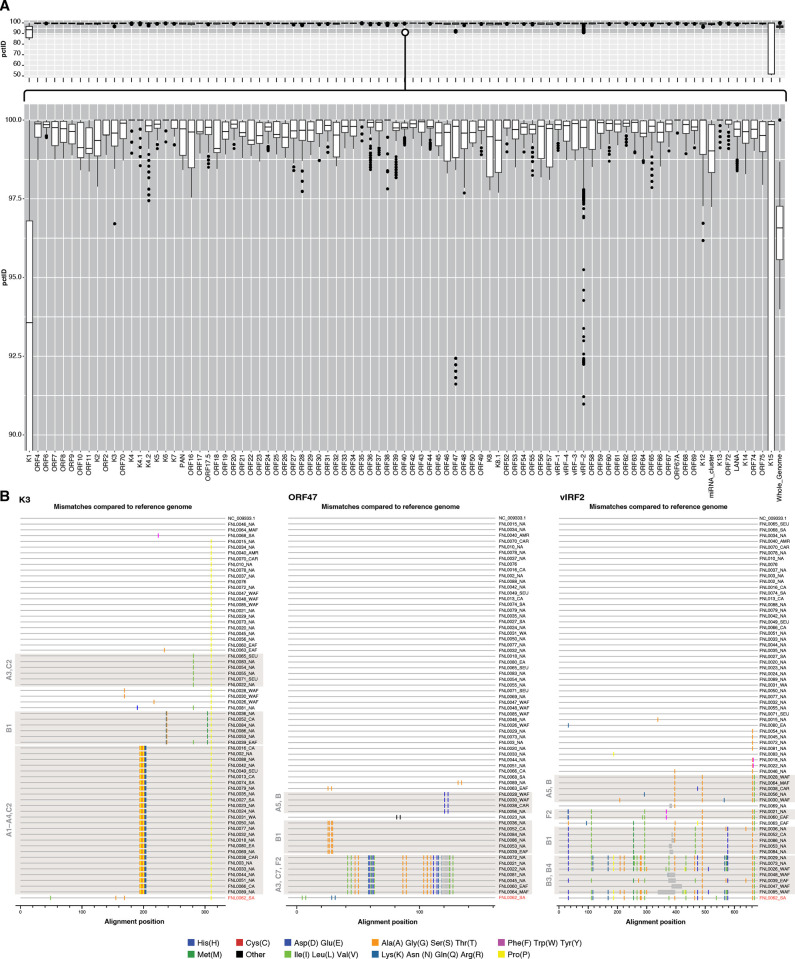
KSHV Percent Identification by Gene Region. **(A)** Boxplots reflect the distribution of pairwise distances calculated for each coding regions across the KSHV genome using nucleotide variation. The distances are based upon a consensus alignment of 66 KSHV genomes from the current study and the NC_009333.1 reference. The highly variable K1 and K15 genes diverge approximately 15% and 50% respectively. The genes within the central portion of the genome are very conserved at 98.5–99.4% with notable exceptions as shown in panel 3B. **(B)** Los Alamos Highlighter amino acids plots illustrating the variations in three gene regions, K3, ORF47, and vIRF2. The plots show the predicted amino acids changes referencing NC_009333.1 shown at the top of the plots. Gaps in sequences are indicated by gray bars.

**Fig 5:**
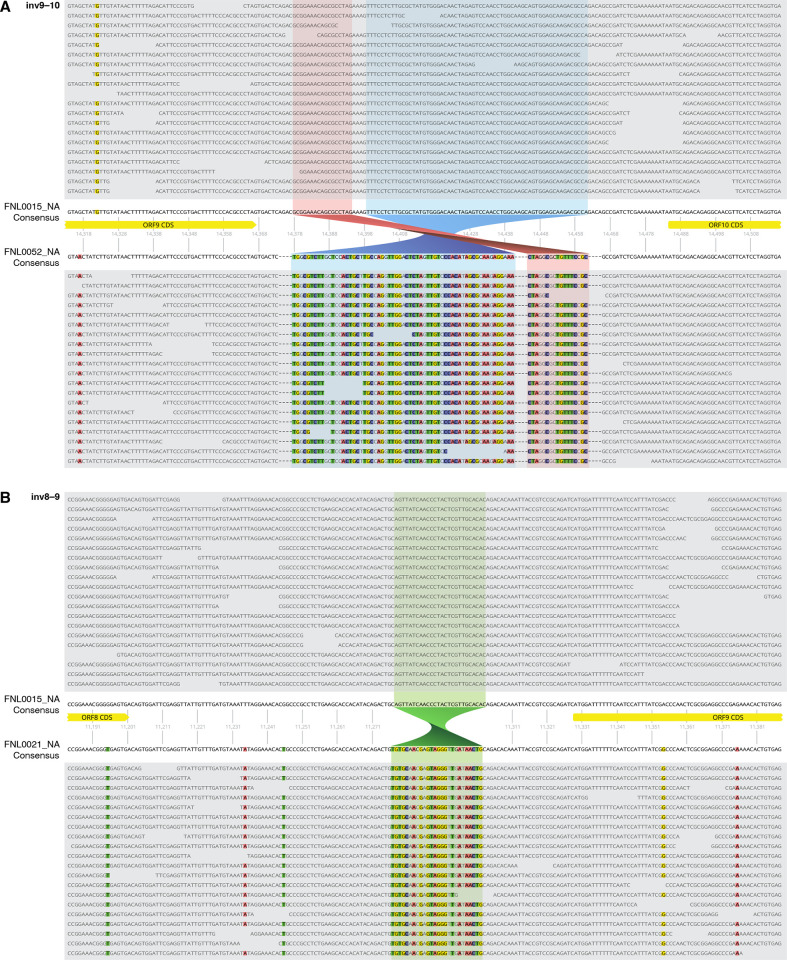
KSHV sequence inversions. **(A)** Reference-guided sequence alignments for samples FNL0015_NA and FNL0052_CA illustrating the ORF9-ORF10 sequence inversion involving 80 base pairs. Sequence deletions occur in FNL0052 accompanying the inversion as shown. **(B)** Reference-guided sequence alignments for samples FNL0015_NA and FNL0021_NA_20050819 depicting the 26bp ORF8-ORF9 sequence inversion. 20X read depth coverage is included for each sample out of mean coverages of greater than 180X for each. Sequence FNL0015_NA is a K1 C3 subtype genome while FNL0052_CA is B1 and FNL0021_NA_20050819 is F2.

Analysis of viral genomes compared to the GK18 reference sequence NC-009333.1, highlights sequence variations in samples from Africa. Within this diverse dataset, variations in the individual coding regions of most genes ranged between 0.3–4%, with exceptions (Fig 4A). The largest variability of ≥ 8% is observed in the K1, K15, ORF47, and the vIRF-2 (K11) genes. Interestingly, variation of ≥ 4% was observed in the miRNA and PAN RNA encoding regions. In comparison, genes with high sequence conservation of ≥ 99.4% across all study genomes included K6, K7, ORF6, ORF35, ORF40, ORF42, ORF61, and ORF67A (Fig 4A). Many previously reported polymorphisms were observed, including early stop codons in the K4.2 and K8 genes and variations within ORF47 and vIRF2 (K11) (Fig 4B) [12,19,20]. Of note, a sequence inversion involving an 80 bp area between ORF9 and ORF10, which includes two deletions (11 bp and 4 bp) and an insertion (3 bp) was identified in five K1 B subtype genomes (Fig 5A).

Due to the rarity of the K1 E and F subtypes, sequence analysis was performed using three recently published F1 and F2 genomes, together with the near full-length E2 and F2 sequences obtained in the current study, to confirm distinguishing subtype-specific polymorphisms [5] (S4 Fig). Novel sequence variations associated with the F subtype include a second inversion polymorphism involving a 26 bp region between ORF8 and ORF9 observed in both F subtype samples, FNL0021_NA and FNL0060_EAF (Fig 5B). This inversion is shared by all published F subtypes but also with additional published sequences from Africa, including B subtypes [20]. The K1 subtype E2 sequence, FNL0062_SA, has both unique variations across the viral genome and previously mentioned polymorphisms in common with B and F subtypes. Of note, a 303-nucleotide deletion in the microRNA encoding region between mir-K12-6 and mir-K12-5 (positions 121190 to 121492 in GK18 reference genome) was exclusively observed in the E2 sequence (S4 Fig). This indel does not include the pre-miRNA sequences for either miR-K12-5 or miR-K12-6, which remain intact, but the deletion would include the pri-miRNA sequence. In addition, insertions were observed within the K15 P allele, comprising 10 bp at the beginning of intron 1 and 33 bp within exon 5 (positions 136675 and 135764 in GK18, respectively). Two different tissues from the same participant were sequenced, confirming these sequence variations. The 10 bp K15 insertion was also observed in the K1 B4 subtype sequence FNL0063_EAF which was likewise obtained from different tissues of the same participant as confirmation. The accumulation of variations within the E2 genome causes it to clusters separately in PCA analysis (Fig 2) and in phylogenetic analyses of the full genome (Fig 1B). An extensive analysis of 284 available KSHV sequences confirmed the indels reported in S4 Fig, except for the B3 subtype-associated deletion in the ORF 4 gene likely due to the few B3 subtype genomes available for comparison (e.g. the ORF8-9 inversion noted in all published F subtype genomes [5] and the ORF9-10 inversion observed in the genomes of several studies [20–22]).

### Functional Polymorphisms found in the E2 Subtype

**Fig 6:**
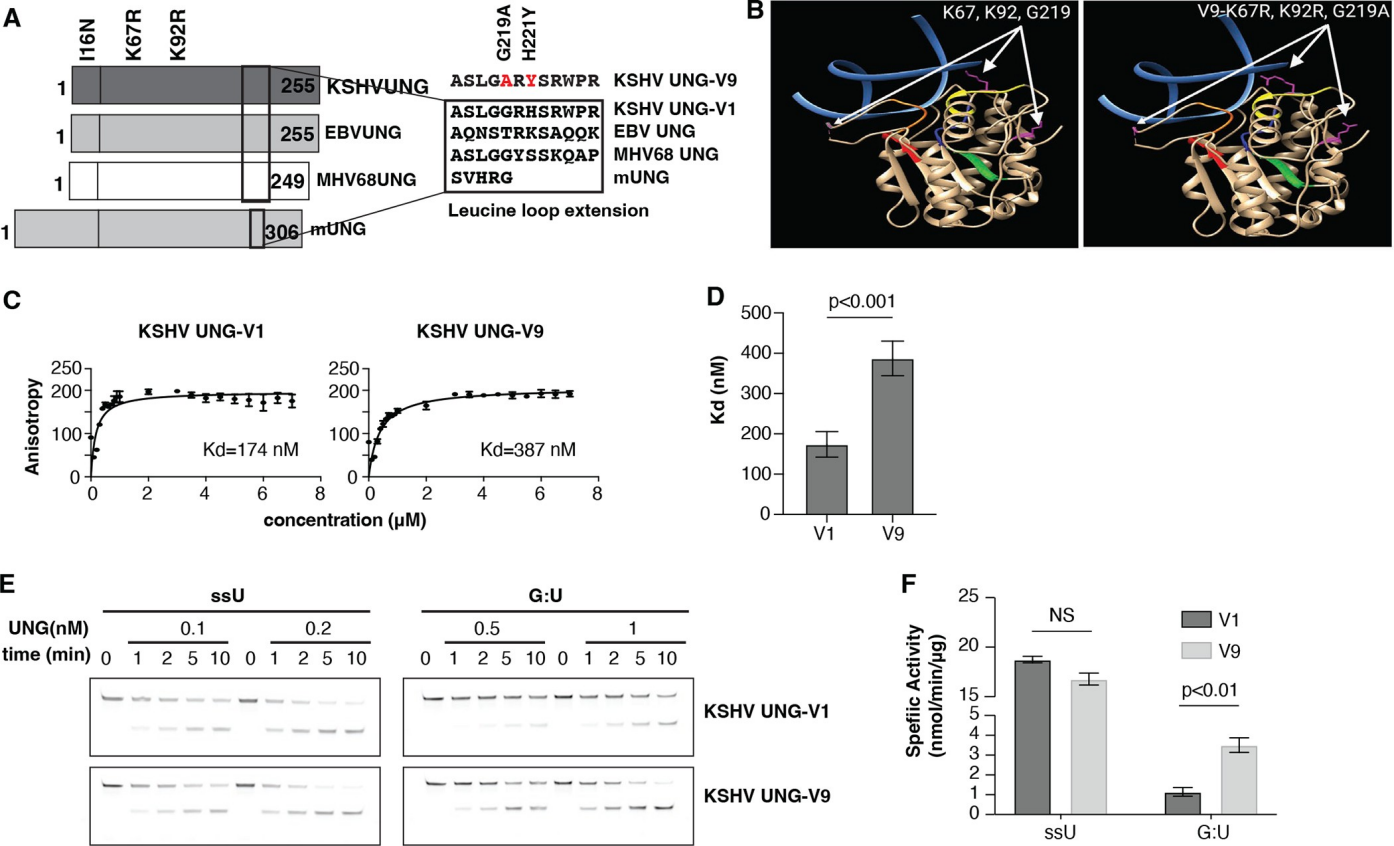
AP site binding and catalytic activity of KSHVUNG variant V9. **(A)** Primary sequence alignment of KSHVUNG and V9 variants, five aa changes in V9 are noted. **(B)** Location of aa changes in the regions resolved for the KSHV vUNG structure (23) using UCSF Chimera. **(C)** Hyperbolic curve for KSHVUNG binding to THF:G-fluorescein-dT dsDNA to plot anisotropy data. **(D)** summary bar graph to compare dissociate constant (Kd) of KSHVUNG and V9 with Extra sum of squares F-test, p<0.001. **(E)** Representative denaturing urea PAGE resolving uracil containing oligo incubated with recombinant KSHVUNG or V9 over time. **(F)** summary bar graph representing specific activity of KSHVUNG and V9 for uracil in ssDNA and G:U mismatch in dsDNA. Unpaired student T-test was used for statistical analysis. NS denotes not significant. Three replicated experiments were performed for Kd and specific activity determination.

As this study is the first to publish a near full-length genome of the K1 E2 subtype, FNL0062_SA, polymorphisms that were distinguishing it from genomes of other KSHV subtypes were mapped. Polymorphisms within ORF46, encoding for the viral uracil DNA glycosylases (vUNG), were particularly notable. Although similar in structure and sequence identity, the viral uracil DNA glycosylases (vUNG) of KSHV and other gammaherpesviruses (GHV UNGs) have notable differences from mammalian UNGs. Structurally, the N-terminal domain of the GHV UNGs is shorter than that of mammalian UNGs, but the GHV UNGs are distinguished by an extended leucine loop, a motif in the DNA binding domain, that is longer than in the UNGs of other herpesviruses and of mammals (Fig 6A) [23]. Regarding biochemical properties, mammalian UNGs process uracil in dsDNA more efficiently than GHV UNGs, but the GHV UNGs have the unique ability to associate with abasic (AP) sites, the end-product of UNG uracil removal. We recently reported that residues in the GHV UNG leucine loop (Fig 6B) were determinants of both dsDNA substrate activity and AP site affinity [24]. The sequence of KSHV ORF46 coding region was generally well-conserved in this study varying only ≤1.5% at the nucleotide level between genomes from different geographical regions (Fig 4A). Thirteen total amino acid (aa) changes were noted in the genome of FNL0062_SA (a KSHV K1 E2 subtype) and led to the grouping of the genomes into nine distinct variant groups, with the reference NC_009333.1 designated as variant 1 (V1) (Table 3).

**Table 3:** Summary of amino acid changes in KSHV ORF46 gene region in 135 current study and available reference genomes.

Unique ORF46 variants	Amino acid change	KSHV Genomes (out of 135)
ORF46-V1	Reference NC_009333.1	34 (25.2%)
ORF46-V2	L26F	11 (8.1%)
ORF46-V3	R53L	3 (2.2%)
ORF46-V4	K67R	55 (40.7%)
ORF46-V5	T247I	1 (0.7%)
ORF46-V6	G12D, K67R	1 (0.7%)
ORF46-V7	T8M, T13S, K67R, K92R	18 (13.3%)
ORF46-V8	Q6K, T13S, S15P, K67R, K92R	11 (8.1%)
ORF46-V9	I16N, K67R, K92R, G219A, H221Y	1 (0.7%)

Six aa changes were noted within the 27 amino acid N-terminal region that influences abasic site binding [24]. A K92R change adjacent to the water-activating loop, a domain key to catalytic activity was noted in three variants. Variant 9 had a total of five amino acid changes including K92R and two changes, G219A and H221Y, in the leucine loop region (Fig 6A and 6B).

The G219A and H221Y changes in the extended leucine loop of KSHV UNG-V9 led us to test for alterations in catalytic activity. To determine if affinity to AP sites was altered in the KSHVUNG-V9, DNA-protein interactions were measured by fluorescent anisotropy using purified recombinant proteins (S5 Fig). This assay is a quantitative measure of protein-DNA interaction in equilibrium. KSHV UNG-V9 had a greater than two-fold increase in the dissociation constant (Kd), indicating a reduction in affinity to abasic sites compared to the prototype KSHV UNG-V1 (Fig 6C and 6D). Next, the impact of amino acid changes in V9 on substrate activity was examined by uracil excision assays. An uracil DNA oligo substrate was incubated with various concentrations of UNG over a time course (Fig 6E). Uracil removal was detected by breakage of the DNA and conversion to a faster migrating form upon hot alkali treatment. No difference was noted in the activity KSHV UNG-V9 on a ssDNA uracil substrate (ssU), however this variant had a significant increase in uracil excision activity on a dsDNA substrate that contained a G:U mismatch (Fig 6E and 6F). This data indicates that coding changes in different KSHV genomes impact enzymatic functions of KSHV vUNG.

## Discussion

The first aim of the current study was to expand the number and diversity of KSHV genomes available to the research community to include diverse subtypes worldwide. This study adds 66 near full-length KSHV genomes of all K1 subtypes except D. KSHV sequences were obtained from individuals with at least one, and often multiple, KSHV-associated diseases. A major strength of the study is the inclusion of patients with all KADs, including KS, PEL, KSHV-LCL, MCD, and KICS, which are likely underdiagnosed due to the diagnosing criteria requiring specialized resources. The study population was diverse, originating from 22 different countries across five continents, making this study unique in the breadth and number of genomes obtained. Using NC_009333.1 (GK18), which is a C3 K1 subtype, as a reference, we observed that genomes of different K1 subtypes appear to have their own conserved polymorphisms; these include the indels within the K1 gene used to identify viral subtypes, but also numerous others across the genome, shared between genomes of the same K1 subtype. (S1 Fig). Analysis of KSHV genomes continues to be restricted by limited number of full and partial genomes currently available, and lack of exemplificative sequences from all subtypes and diverse geographical regions. Few or no KSHV sequences from subtypes D, E, and F are published. Distinctive polymorphisms in KSHV genomes from the Pacific rim, Asia, including the E subtype have been identified previously in the variable K1 and K15 genes as well as genes within the central portion of the viral genome [25] via targeted sequencing. While the near full-length K1 E2 genome sequence reported here (FNL0062_SA) is the only one currently available, the previous targeted sequencing of specific variable genes in D and E subtypes allows limited comparisons. The distinctive K15 P allele insertions noted in FNL0062_SA were previously reported including insertions of 27 nucleotides (9 amino acids) within exon 5 in two Polynesian D and a Hualien E3 subtype from Taiwan. The E2 sequence, FNL0062_SA, has a similar insertion but with an additional 2 amino acids. FNL0062_SA also shares the same 10 bp insertion at the beginning of K15 intron 1 previously observed in B subtypes, including the B4 subtype sample FNL0063_EAF in the current study [25].

Next, we assessed intra-host viral sequence variations. Whenever possible, we analyzed parallel viral libraries created using different tissues/ biological fluids from the same individual including PBMC, oral fluids, BAL, KS biopsies, and effusions. This was also part of the quality control procedures to ensure data integrity (S1 Table). In 17 of 18 tissue comparisons, no substantial sequence variations were observed that could not be explained by differences in read depth coverage, known artifacts in llumina sequencing [26], or expected alignment problems near repetitive and homopolymer regions of the viral genome. However, due to the high conservation of KSHV genomes observed within our study and across independent samples (S4 Table), we cannot exclude the possibility that multiple viruses with very similar genomes infecting the same individuals were unresolved. One exception was noted in FNL002. For this individual, it is unclear if the polymorphisms observed are the result of within-host evolution of the same genome or are the result of an underlying multiple KSHV infection of two very similar K1 A subtypes (Figs 1B and S2). It should be noted that a multiple infection was identified in sequential sequencing of three longitudinal PBMC samples from FNL0021. While the earliest KSHV genome was a K1 subtype C3, in two subsequent samples taken five and 12 months later, a predominant subtype F2 was sequenced with low levels of polymorphisms suggesting a co-infection with a K1 C genome (S3C Fig). Unfortunately, the genome sequence obtained from an effusion sample of FNL002 in 2020 was determined using a shotgun protocol not compatible with our variant analysis pipeline. The read depth coverage using shotgun sequencing is also substantially lower than targeted sequencing using custom baits, further complicating confirmation of mixed infections [15].

As we had very few available tissue biopsies in this study, our findings did not replicate a recent report comparing KSHV genome sequences from matched KS biopsies and oral fluid samples from Uganda [19] which found indels and large structural variations within genomes from KS lesions. This study included only tissues with KSHV viral loads within the optimal genome copy range of our sequencing process. We acknowledge that preferential sequencing of samples with higher viral load limits the inferences afforded by our current method, thus a thorough comparison between the studies is not possible. Detection of multiple infections within participants also complicated sequence comparisons.

However, a secondary benefit of repeated sequencing and inclusion of various biological samples was the confirmation of multiple infections in 5 individuals (Fig 1 and S1 Table). Three factors could result in samples falsely appearing as harboring multiple-infection: (1) Index-hopping, (2) contamination during sample processing and library preparation, and (3) sequencing error. For 11 of 14 identified multiple infections, validation was performed at the molecular level via Sanger sequencing using subtype-specific primers, as previously described [12,15]. Areas distinguishing the subtypes were identified for forward primer design while a conserved region was used for the reverse primer (S3 Table). When possible, DNA from the original sample was used to confirm multiple infections. Otherwise, additional samples from the same individual were used. Successful confirmation of multiple K1 subtypes via Sanger sequencing was dependent upon both the relative viral load of the minor variant within the sample, and the level of sequence variation between subtypes constituting the multiple infection. For example, FNL0025_NA and FNL0071_SEU, with multiple infections of subtypes C1 and C2, were more difficult, but ultimately successfully resolved via Sanger sequencing, due to the high degree of similarity between the subtypes. These were further resolved using our *de novo* assembly pipelines, which features independent assembly of K1 and K15 gene regions, in addition to full-length assembly (S1 Document). In such cases, complete contigs for each K1 subtype were successfully generated.

Random error is a known phenomenon in high-throughput sequencing, and a certain level of background noise is expected and acceptable in any given sequencing run. Multiple infections can be hard to distinguish from sequencing error when the minor genome is present at low frequencies. We thus sought to develop a method to highlight the presence of multiple infections where the frequency of the minor genome was significantly lower than the dominant genome or in cases where additional sample material was limited or unavailable. Thus, our observations of multiple genome variants within one sample were confirmed by: the assembly of more than one complete K1 and/or K15 subtype specific contig, subtype-specific Sanger sequencing, and the observation of a pattern of validated variant sites occurring at similar relative proportion across the genome as reported in Table 2. It should be noted that participant FNL0090, for which subsequent PBMC specimens showed decreasing frequency of the minor variant, was receiving treatment which resulted in a deceased PBMC KSHV viral load between sample collections from approximately 2,080,000 to 364,000 copies per million cells. [27,28] Many factors contribute to detection of multiple variants including the method and amount of DNA used for library construction, data analysis and manual inspection of overlapping reads in variable gene regions, and, importantly, the read depth coverage. This study design does not inform as to how a multiple infection occurs in an individual, whether as sequential infections or as one episode of transmission with a swarm of viral genomes. The study similarly cannot inform as to whether HIV contributes to multiple infections, as most participants were living with HIV. As technologies for viral sequencing improve, dependency upon high viral copies will decrease allowing the expansion of observations to include other populations. We also documented multiple infections in a recent study in Cameroon, which is presumably related to the high seroprevalence of KSHV within the population, the high frequency of KSHV DNA detection and high VL [12]. The fact that multiple infections do occur however, is an important consideration for future vaccine development.

A third goal of the study was to examine sequence variations across the viral genomes potentially associated with the development of various KAD. KSHV variations and disease associations have been investigated previously due to the unique subtype distribution of KSHV and regional differences in the reported incidence of associated cancers. Several studies examining KSHV K1 subtype and disease have reported associations with risk or severity of KS [8,29]. Analysis has been recently extended to whole viral genomes with advances in next-generation sequencing technologies [5,12,19,30]. Few studies have looked at near full-length genomes except for Jary et al. who reported that the F2 subtype is associated with severe disease in cases of MCD [5]. Our recent analysis of KSHV genomes in Cameroon did not find sequence differences between matched cases and controls that could be associated with KS [12]. We have previously observed that certain combinations of polymorphisms within the pre and mature miRNA encoding regions are associated with the development of MCD [18] with the most informative polymorphism, NC_009333.1:g.118004A>G, identified within the K12 region. The combinations of loci previously determined to be associated with MCD were observed in similar frequencies in the current study. Although these variations appear frequently in MCD and KICS cases, they are also observed in individuals with no history of MCD or KICS. However, analysis is complicated due to the observation that individuals with MCD often have histories of other KAD. In addition, insufficient numbers of sequences from the microRNA region are available from individuals without disease to allow the proper assessment of the contribution of sequence to risk of disease. Whole virus genome sequencing efforts are underway to address this discrepancy. In this study, a PCA analysis of 45 individual genomes with varied disease histories did not identify a specific clustering of disease type segregated by viral sequence variations. A total of 1989 variations spanning the viral genome were examined, including both coding and non-coding regions, with only repetitive regions excluded. The PCA indicated that samples cluster mainly by K1 subtype. As mentioned, complicating the analysis was the complex histories of multiple KAD of most study participants. Investigation of the contribution of KSHV sequence to disease risk would require sequencing of more KSHV genomes from individuals without KAD, as previously observed. Similar studies in different clinical and epidemiological context would be necessary to generalize this finding to other populations. The contribution of HIV to outcomes also cannot be adequately addressed due to the study design. Epstein-Barr virus sequence (EBV) variations associated with the risk of nasopharyngeal carcinoma and Burkitt’s lymphoma have been reported due to concentrated sequencing efforts resulting in substantially larger numbers of EBV genomes available for analysis [31,32]. As KSHV genomes sequences continue to be generated, variations may be identified that will be informative and provide drug targets for prevention and treatment of disease.

While complex sequence variations within subtypes were highlighted in the current study, even single nucleotide polymorphisms can affect protein expression as exemplified by the functional consequences of SNVs within the ORF46 gene. In this study, we biochemically characterized DNA binding and catalytic properties of the natural variant KSHVUNG-V9, encoded by a variant ORF46, observed in the K1 E2 subtype (FNL0062_SA). We found it has reduced affinity for AP sites and gained specific activity on G:U dsDNA substrates. Two of the variant amino acid changes are in the leucine loop extension, a unique structure in the DNA binding domain of UNGs. The leucine loop extension in mammal UNG is 5 amino acids long, and functions to flip out the uracil base in dsDNA during catalysis [33]. In GHV UNGs the leucine loop extension is 12 residues long and has additional properties. These include AP site affinity, altered activity of dsDNA substrates [24] and structurally, the ability to flip out the widowed base in a dsDNA [23]. Two mutations in the V9 (G219A, H221Y) are within the leucine loop extension. Deletion of the leucine loop extension or alteration of specific residues affect catalytic function and AP site affinity [24]. Therefore, we hypothesized that the KSHVUNG-V9 would have altered biochemical properties. Our data clearly show that AP site affinity is decreased. While the physiologic role of AP site binding has not been determined, we hypothesize that the lower activity on dsDNA in GHV UNGs versus mammal UNGs may be related. AP sites are the end-product of uracil glycosylation and continued occupancy by UNG would limit turnover. With V9, the lower AP site affinity may drive faster release following catalysis that is reflected by the increased dsDNA substrate activity. Future studies may examine whether the KSHVUNG-V9 conveys a protective advantage in environments where high levels of genomic uracil are generated, such as with expression of the APOBEC cytidine deaminases. [34]

The overall effect of the numerous polymorphisms identified in diverse viral genomes are beyond the scope of the current study, but it is hoped that the expanded sequence information will provide meaningful insights into KSHV biology. The sequence inversions observed between the ORF 8–9 and ORF9-10 coding regions in genomes from Africa have not been previously reported but, upon review, they are identifiable in other published KSHV genomes [5,20,22,30]. Inversions can result from recombination and have been shown to be increasingly common in the human genome [35]; they can have wide-ranging effects, from imperceptible to potentially serious, and have been associated with human diseases including hemophilia [36] and, increasingly, in other diseases [37,38]. Sequence inversions have been described before in Epstein-Barr virus [39]. Functional consequences of the newly identified inversions are unknown, as they occur outside coding regions, but each could potentially affect the protein expression of nearby genes particularly in the case of the ORF9-10 inversion which results in the loss of sequence (Fig 5). Additional notable sequence variations observed in the current study are summarized in S4 Fig and include the 11 amino acid indel in the K3 gene, one of the two E3 ubiquitin ligases encoded by KSHV, which was detected in 27 of the current study genomes of K1 A and C subtypes. This indel was noted in GenBank direct submissions by the Hayward laboratory in 2018 and is particularly interesting as it has not been found in any samples of individuals of Africa descent which suggests that it is an adaption acquired by KSHV after humans migrated out of Africa. K3 functions to down-regulate MHC-I in late infection and has been shown to be important in modulating the host antiviral response [40]. The biological consequences of the insertion are unknown but warrant further investigation.

While polymorphisms over-represented in genomes from Africa are of particular interest due to the high incidence of KAD in this region, it should be noted that sequences in the current study were all derived from participants with history of disease originating from 22 different countries. Future studies are planned to examine the contributions of sequence variation to outcomes such as severity of disease. We are actively pursuing the sequencing of additional samples from isolated populations, people without histories of KAD, and from individuals living without HIV. In addition, functional studies are ongoing investigating polymorphisms identified in genes of interest and within the non-coding RNA regions. The major objectives of the study were met as we substantially increased both the number and diversity of near full-length KSHV genomes sequences available to the research community including the first known near full-length sequences of genomes of the K1 C7 and E2 subtypes. Additionally, polymorphisms and structural variants of interest were identified, some of which appear to be K1 subtype and/or geographically defining. Notably, the identification of KSHV multiple infections within persons with KAD has potential implications for treatment decisions and future vaccine development.

## Materials and methods

### Ethics statement

Protocols for the collection, storage, and processing of samples were approved by the National Cancer Institute Institutional Review board and all participants gave written informed consent according to the Declaration of Helsinki.

### Study design and sample collection

Oral fluid, PBMC, KS and lymph node biopsies, bronchioalveolar lavage, CSF and effusion samples were collected from 78 individuals, enrolled in clinical trial 01-C-0038 (NCT00006518) from 2000–2022, at the National Cancer Institute (NCI) HIV and AIDS Malignancy Branch (HAMB), Bethesda, MD, USA; all but nine were people living with HIV. Trial participants with KAD are referred to the HAMB from institutions worldwide. To protect personally identifiable information, United Nations guidelines for geographical designations were used to describe the region of origin.

### Sample processing, and viral genome sequencing

DNA was extracted from all 102 samples, excluding tissue biopsies, using Qiagen’s blood and body fluids mini kits as previously described [41,42]. Tissue samples were processed using Trizol (Thermo Scientific) [17]. Samples included in the study were selected according to KSHV viral load measured by real-time qPCR as previously described [12]. The VOS procedures for processing both blood and effusion samples and the KSHV qPCR assay itself are certified by the Clinical Laboratory Improvement Amendments (CLIA) [43]. Separate dedicated rooms are used for reagent preparations, sample processing, DNA extractions and qPCR assay setup, qPCR amplification, library preparations, and Illumina sequencing to prevent contamination. Briefly, samples with estimated >6,000 copies per library input, were processed for NGS using either the 200 ng or 3 μg Agilent SureSelect XT Custom library preparation protocol (Agilent Technologies, Santa Clara, CA). Our custom bait design covers the long unique region of the KSHV genome as well as all known KSHV K1 and K15 subtypes, excluding the internal repeat regions and terminal repeats. Each library was labeled with a unique barcode identifier to allow pooling of samples. Samples for the comparisons of alternative tissue from the same individual were not pooled together but sequenced separately (S1 Table). All libraries were pair-end sequenced using 500 cycle V2 cartridges on Illumina MiSeq instruments (Illumina, Hayward, CA) [12]. To distinguish Illumina errors from mixed viral infections, pellets from a VG-1 single colony were used to determine substitution error probabilities as described in S1 Document. In short, 200 μL of a VG-1 cell suspension at 2*10^4^ cells/mL were plated in two serial dilution series across a 96 well plate. Detectable single colonies were sub-cultured from wells into larger vessel prior to being pelleted at 8000 x g and DNA was extracted as described above.

### Reference-guided and de novo assembly pipeline

For each sample, NGS reads were assembled using both a reference-guided alignment method previously published [12] and a newly developed *de novo* assembly protocol optimized in house. The details of the *de novo* method are available as S1 Document. Any sample with less than 30X median read depth coverage or < 98% reference base coverage was excluded from analysis, the results of which are shown in S2 Table. Five areas of repetitive sequence were masked in the final alignments (NC_009333.1:g.24230-25045, 29927–30055, 118229–113914, 124784–126456, and 137169–137969). Sample consensuses generated by both pipelines were utilized to finalize consensus alignments including confirmations of deletions and unique sequence variations as well as resolution of misalignments within variable gene regions. Completed curated consensus sequences were exported as FASTA files from Geneious (Geneious Prime 2022.0.2) for downstream analyses and submission to GenBank.

### Tissue/Compartment comparisons

For 26 individuals, KSHV genomes were successfully sequenced from multiple tissues including PBMCs, oral fluids, effusions, biopsies, bronchioalveolar lavage, and lymph nodes. In each comparison, NGS libraries were prepared independently and were not combined in the same pool during Illumina runs (S2 Table). In two cases, comparisons involved previously published KSHV genomes derived from effusions compared with a different material in the current study [15]. Pairwise alignments between sample consensus sequences from each material type were generated in Geneious (Geneious Prime 2022.0.2), inspected for variations, and percent identities were calculated (S1 Table).

### Phylogenetic analyses

Consensus near full-length and K1 specific sequences were aligned with published KSHV genomes in Geneious (Geneious Prime 2022.0.2) using the MAFFT algorithm with default settings. The resulting K1 sequence alignment was exported from Geneious and used to infer phylogenetic trees using IQTree version 2.2.0.5 with default settings using 1000 bootstrap replicates (http://iqtree.cibiv.univie.ac.at/) [44]. IQTree output files were visualized in FigTree version v1.4.4 (http://tree.bio.ed.ac.uk/software/figtree) [45]. K1 gene subtype was determined based upon similarity to published KSHV K1 sequences. The multi-genome sequence alignment, was visualized in SplitsTree [46]. The Neighbor-Net method of phylogenetic tree construction was used to create a splits network generated using default settings with 1000 bootstrap replicates. A Pairwise Homoplasy Index (PHI) test was performed to assess recombination within the resulting network in SplitsTree v4.15.1 [47]. Samples determined to have KSHV mixed infections were not included in the near full-length phylogenetic tree construction or subsequent phi test analysis. K15 gene subtypes reported in S2 Table were determined by sequence homology using Blast [48]. The SplitsTree sequence alignment was used to calculate percent identity between individual KSHV genomes within Geneious software which is presented as a distance table (S4 Table). The identity table used 131,256 bp of sequence excluding the internal repeat regions (NC_009333.1:g.24230-25045, 29927–30055, 118229–113914, 124784–126456, and 137169–137969).

### Mixed infection verification and analyses

Potential mixed infections are observable *in silico*. In reference-guided alignments, BAM files are manually evaluated using Geneious (Geneious Prime 2022.0.2) for genome regions with a significant portion of reads exhibiting patterns of single-nucleotide polymorphisms (SNPs) compared to the majority consensus. In addition, our de *novo* assembly protocol features optional targeted subassembly of K1 and K15 gene regions by prefiltering sequence data against 19 K1 and three K15 published sequences representing all known subtypes (S1 Document). In cases of potential mixed infection, we observe complete assembly of multiple K1 and/or K15 subtypes within a single sample. For samples with multiple KSHV genome subtypes detected, we applied a binomial test process to distinguish and validate nucleotide variance occurring more frequently than would be expected due to random error. Expanded details of mixed infection analysis are available in S1 Document. For each sample with putative multiple infection, K1 subtype specific contigs were used to design custom primers for Sanger sequencing as previously described [12,15]. When possible, DNA from the original sample was used to confirm multiple infection, otherwise, samples from the same individual from other dates were used. Validated K1 gene region sequences from samples with multiple infection were then used to construct phylogenetic trees for subtype identification as previously described using IQTree (Fig 1A). For samples with multiple K1 subtypes verified by Sanger sequencing, we applied a binomial test process to distinguish genome-wide nucleotide variance occurring more frequently than would be expected due to error, which allowed detection and visualization of relative proportions of putative SNPs detected across the genome (Expanded detail in S1 Document). Haplotyping and other variant calling techniques were not feasible using short-read high throughput sequencing due to limitations imposed by the genome structure of the virus, i.e. large regions of high homology. For longitudinal samples from two participants (FNL0021 and FNL0090), the reads from each sample were mapped to the consensus from the initial time point. For sequences obtained from different tissues from the same participant (FNL008, FNL0039, FNL0059, FNL0067, FNL0071, FNL0082), the data set with the earliest date or highest sequencing depth was used to generate the sample consensus. The sequence coverage of the major and minor genome sequences was calculated in R by averaging the base coverage of all variant nucleotide positions for the reference and variant nucleotides [49].

### Sequence identity by KSHV gene

We identified the variations within every KSHV CDS region as previously described [12]. Briefly, after curating the consensus sequence of all samples, subalignments of all coding regions (CDS) were created, pairwise distance matrices were calculated, and the results were exported from Geneious (Geneious Prime 2022.0.2). Boxplots generated from the distribution of pairwise distances for each CDS were produced in R.

### KAD and other individual characteristics

A reference-based multiple sequence alignment of near full length consensus sequences against NC_009333.1 was generated using MAFFT with default settings and converted to a variant call format (VCF) using JVarkit (msa2vcf) [50]. This output provided observed nucleotide call counts per *alignment* position with respect to NC_009333.1. These were converted to *genomic* position counts via custom python script to account for alignment gaps. Multi-allelic variants were split, normalized, and assigned unique names using bcftools [51] *norm* and *annotate*. Further, repeat regions and the K15 CDS were excluded using bcftools *view*. Variants without alternate genotypes were excluded using vcftools

[52] [53]. Variants were included even if infrequent as to not exclude subtype variations observed in the rare E and F viral genomes. Of 5005 variants, a total of 3016 variants were excluded based on physical distance of adjacent markers (50 bp with a 5 bp sliding window) and linkage disequilibrium (R^2^ > 0.6) in PLINK v1.90b6.21 (https://zzz.bwh.harvard.edu/plink/cite.shtml). Based on their identity by descent, 21 genomes were excluded (Pi_HAT = 1), leaving one from each closely related pair. Principal components were calculated using 1989 informative variants across 1788 positions from 45 study genomes in PLINK. The first two eigenvalues were plotted in R (Fig 2) [54].

### Biochemical characterization of KSHVUNG activity

Expression and purification of recombinant KSHVUNG used for analysis as well as the UNG enzymatic activity assay were previously described [24]. A single or double stranded oligo containing an uracil was incubated with KSHVUNG and then subjected to hot alkali breakage. The oligo was then separated via denaturing urea gel electrophoresis and quantitatively imaged. Enzymatic activity was measured by the degree of oligo conversion to the faster migrating form. AP site affinity was determined by fluorescence anisotropy analysis. KSHVUNG binding to a dsDNA oligo containing a Tetrahydrofuran: G (THF:G) mismatch, a stable analog of an AP site, was measured. The dissociation constant (Kd) was reported as the concentration of UNG at which half of the total THF:G dsDNA was bound. Additional details in S2 Document.

## Supporting information

S1 Fig Patterns of variant frequency across the KSHV genome by K1 subtype. Variant positions are mapped across the KSHV genome and colored by K1 subtype. Each circle represents a variant position identified at that genomic coordinate. The larger the circle the greater number of samples sharing the variation. Subtypes C7, E, and F were observed in one or a few participants each but were included to illustrate that polymorphisms are frequent outside of the K1 variable gene.
(TIF)

S2 Fig Analysis of KSHV K1 observed in multiple tissue comparisons. K1 multiple tissue alignments in Geneious. K1 polymorphisms confirming subtypes in multiple tissue comparisons and expanded SplitsTree analysis incorporating all multiple tissue genomes. Geneious alignments of the K1 and partial ORF4 genes illustrating the pattern of polymorphisms. Polymorphisms within this variable region are indicated by black bars. The patterns shown are used in proxy of the full genome to show that 17 of 18 multiple tissue comparisons are near 100% identical. Phylogenic analysis of the near-full length genomes is shown in panel B. FNL002, shown first, has polymorphisms suggestive of a KSHV A2 and A4 subtype and may represent a mixed infection but additional longitudinal sequencing would be required to confirm.
(TIF)

S3 Fig Genome variant analysis. For all graphs, the sample-specific reference (major) KSHV genome is shown in blue and the minor genome variant(s) in orange. Each dot represents a position in the genome where a non-reference base distinguishes the minor from the major genome nucleotide at higher rate than expected due to background error. Samples with K15 P and M alleles cannot be resolved beyond the ORF75 gene region by reference-guided alignment. (A) Determination of the minor variant frequency for longitudinally sequenced samples FNL0090_NA which had evidence of multiple KSHV genomes. Three distinct K1 subtypes (A4, B1 and C3) were detected in the PBMC sample from 01 April 2019 while in the PBMC sample collected 24 April 2019 only the A4 and B1 K1 subtypes were detected. In blue are the variant positions at which the sample-specific nucleotide is present at >80% in each sample. On 01 April 2019, variant sites (in orange) occurring at two relative proportions across the genome, one at a > 10% frequency with the other < 5%, were detected. In the sample collected on 9/24/2019, only one such distribution is observed. (B) Mixed infection samples FNL0025_NA, determined to have four concurrent KSHV K1 subtypes a single sample (C1, C2, B1 and C3), and FNL0043_NA, in which two K1 subtypes were detected (C3, B1). (C) Sequencing of longitudinal PBMC samples from individual FNL0021 in which only one KSHV K1 subtype. (C) was initially observed in the earliest time point, noted by a lack of variant positions plotted across the genome. In two later PBMC collections, multiple genome variants predominantly of the K1 F2 subtype and a minor C subtype were identified. (D) Visualization of genome variants sequenced across multiple tissues or compartments for, FNL0039 and FNL0059 (K1 subtypes B4, A4, C3 and C7, B1 respectively). Similar frequency patterns across the minor and major genome variants could be distinguished in both material types.
(TIF)

S4 Fig Indels Table: Positions of notable indels observed within the current study with subtype associations. The positions indicated reference the NC_009333.1 (GK18) genome which is shown at the top of each feature. The summary does not represent all the numerous variations observed in the data set.
(TIF)

S5 Fig Protein quantification using SDS-PAGE. Recombinant UNGs were resolved by SDS-PAGE and stained with Coomassie brilliant blue R-250. Pre-stained protein ladder and serially diluted BSA standard were used for protein size estimation and quantification of recombinant KSHV UNGs.
(TIF)

S1 Table Multiple tissue type comparison summary. Samples used in phylogenetic and PCA analyses are indicated in individual columns. Those sequences for which consensus sequences were submitted to GenBank and for whom short read data has been made available in SRA are also specified.
(DOCX)

S2 Table NGS Statistics. (DOCX)

S3 Table Sanger Primer Designs. (DOCX)

S4 Table Identity table for all KSHV genomes used in SplitsTree analysis (Fig 1B). The identities were determined in Geneious software from an alignment of all samples included in the SplitsTree analysis. The alignment used for identity comparison calculations was 131,256 bp long and excluded the internal repeat regions (NC_009333.1:g.24230-25045, 29927–30055, 118229–113914, 124784–126456, and 137169–137969).
(PDF)

S1 Document Observed per-base mapping frequencies. (DOCX)

S2 Document Observed 95^th^ quantile substitution rates. (PDF)
