# Supplementary figures and images for: Sequencing of Kaposi’s Sarcoma Herpesvirus (KSHV) genomes from persons of diverse ethnicities and provenances with KSHV-associated diseases demonstrate multiple infections, novel polymorphisms, and low intra-host variance
Source: PLoS Pathog. 2024 Jul 15;20(7):e1012338. doi: 10.1371/journal.ppat.1012338 (PMC11271956; doi:10.1371/journal.ppat.1012338)

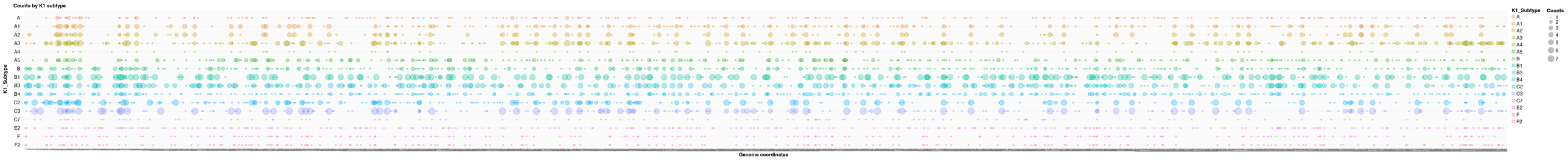

Supplement: S1 Fig — Variant positions are mapped across the KSHV genome and colored by K1 subtype. Each circle represents a variant position identified at that genomic coordinate. The larger the circle the greater number of samples sharing the variation. Subtypes C7, E, and F were observed in one or a few participants each but were included to illustrate that polymorphisms are frequent outside of the K1 variable gene. (TIF) [file ppat.1012338.s001.tif]

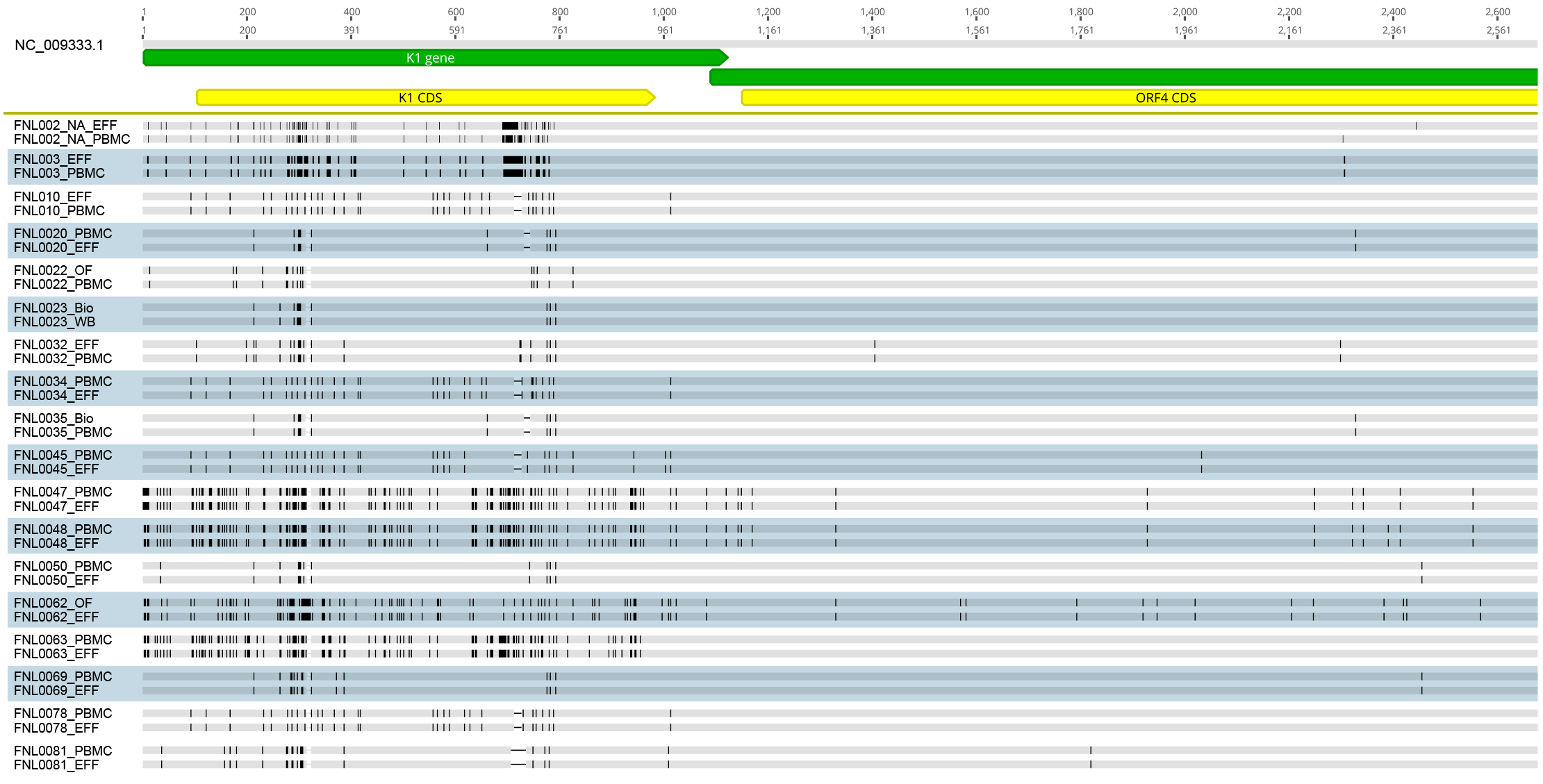

Supplement: S2 Fig — K1 multiple tissue alignments in Geneious. K1 polymorphisms confirming subtypes in multiple tissue comparisons and expanded SplitsTree analysis incorporating all multiple tissue genomes. Geneious alignments of the K1 and partial ORF4 genes illustrating the pattern of polymorphisms. Polymorphisms within this variable region are indicated by black bars. The patterns shown are used in proxy of the full genome to show that 17 of 18 multiple tissue comparisons are near 100% identical. Phylogenic analysis of the near-full length genomes is shown in panel B. FNL002, shown first, has polymorphisms suggestive of a KSHV A2 and A4 subtype and may represent a mixed infection but additional longitudinal sequencing would be required to confirm. (TIF) [file ppat.1012338.s002.tif]

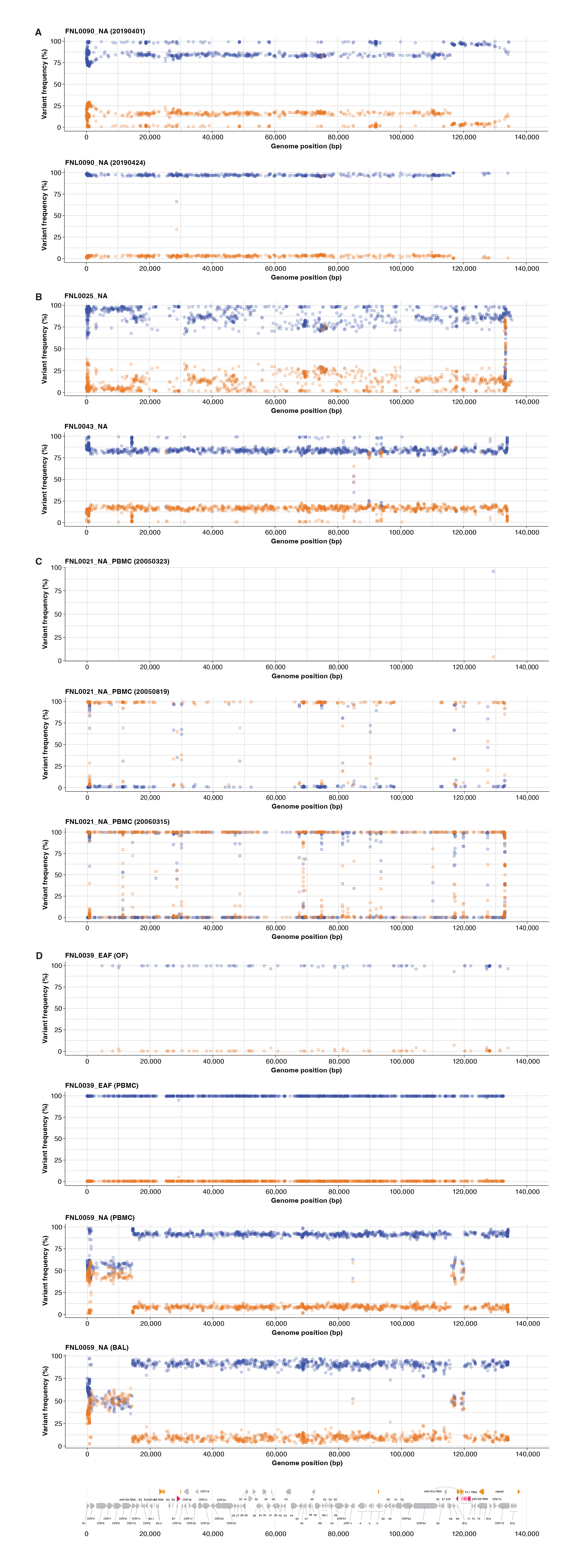

Supplement: S3 Fig — For all graphs, the sample-specific reference (major) KSHV genome is shown in blue and the minor genome variant(s) in orange. Each dot represents a position in the genome where a non-reference base distinguishes the minor from the major genome nucleotide at higher rate than expected due to background error. Samples with K15 P and M alleles cannot be resolved beyond the ORF75 gene region by reference-guided alignment. (A) Determination of the minor variant frequency for longitudinally sequenced samples FNL0090_NA which had evidence of multiple KSHV genomes. Three distinct K1 subtypes (A4, B1 and C3) were detected in the PBMC sample from 01 April 2019 while in the PBMC sample collected 24 April 2019 only the A4 and B1 K1 subtypes were detected. In blue are the variant positions at which the sample-specific nucleotide is present at >80% in each sample. On 01 April 2019, variant sites (in orange) occurring at two relative proportions across the genome, one at a > 10% frequency with the other < 5%, were detected. In the sample collected on 9/24/2019, only one such distribution is observed. (B) Mixed infection samples FNL0025_NA, determined to have four concurrent KSHV K1 subtypes a single sample (C1, C2, B1 and C3), and FNL0043_NA, in which two K1 subtypes were detected (C3, B1). (C) Sequencing of longitudinal PBMC samples from individual FNL0021 in which only one KSHV K1 subtype. (C) was initially observed in the earliest time point, noted by a lack of variant positions plotted across the genome. In two later PBMC collections, multiple genome variants predominantly of the K1 F2 subtype and a minor C subtype were identified. (D) Visualization of genome variants sequenced across multiple tissues or compartments for, FNL0039 and FNL0059 (K1 subtypes B4, A4, C3 and C7, B1 respectively). Similar frequency patterns across the minor and major genome variants could be distinguished in both material types. (TIF) [file ppat.1012338.s003.tif]

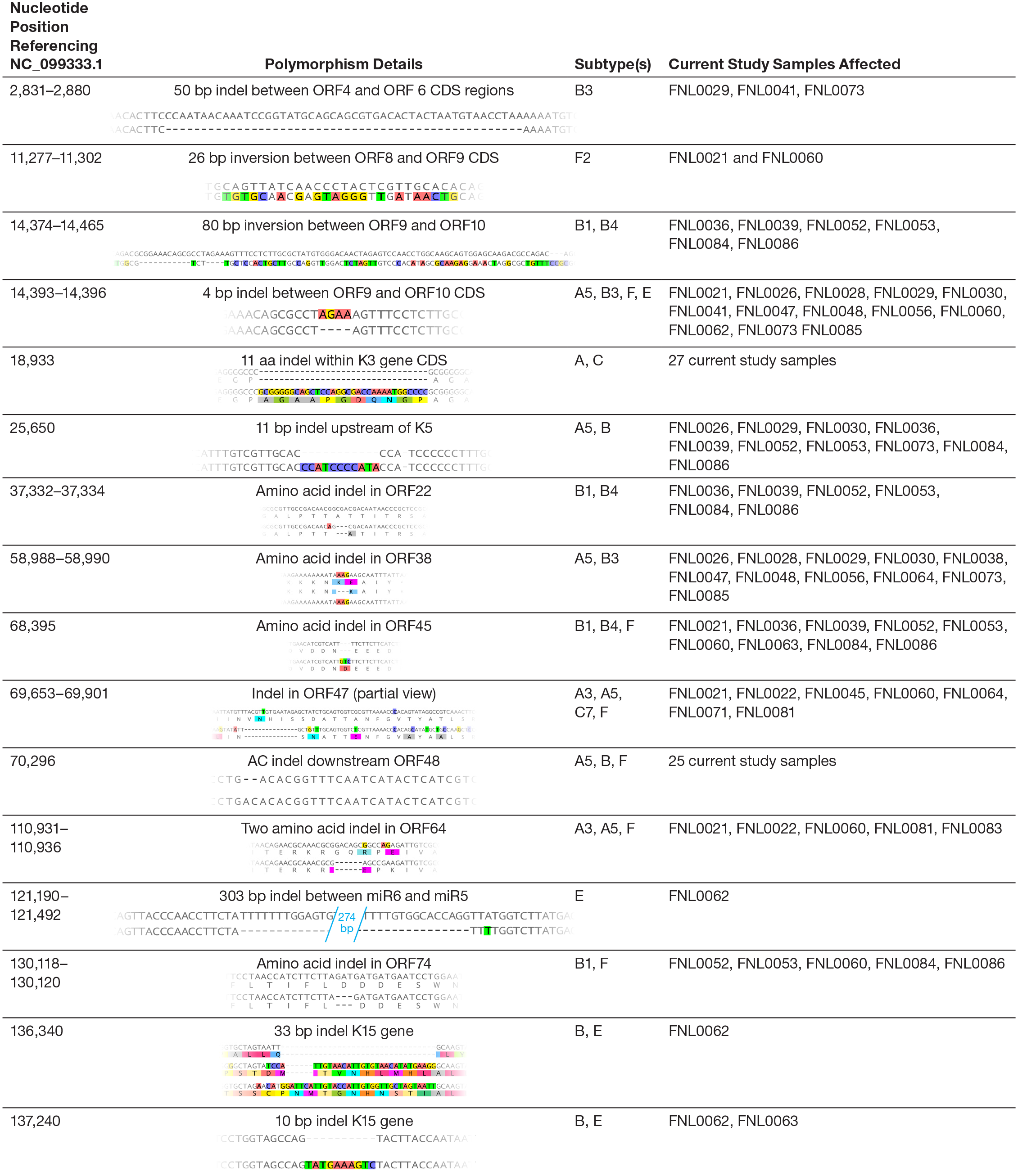

Supplement: S4 Fig — The positions indicated reference the NC_009333.1 (GK18) genome which is shown at the top of each feature. The summary does not represent all the numerous variations observed in the data set. (TIF) [file ppat.1012338.s004.tif]

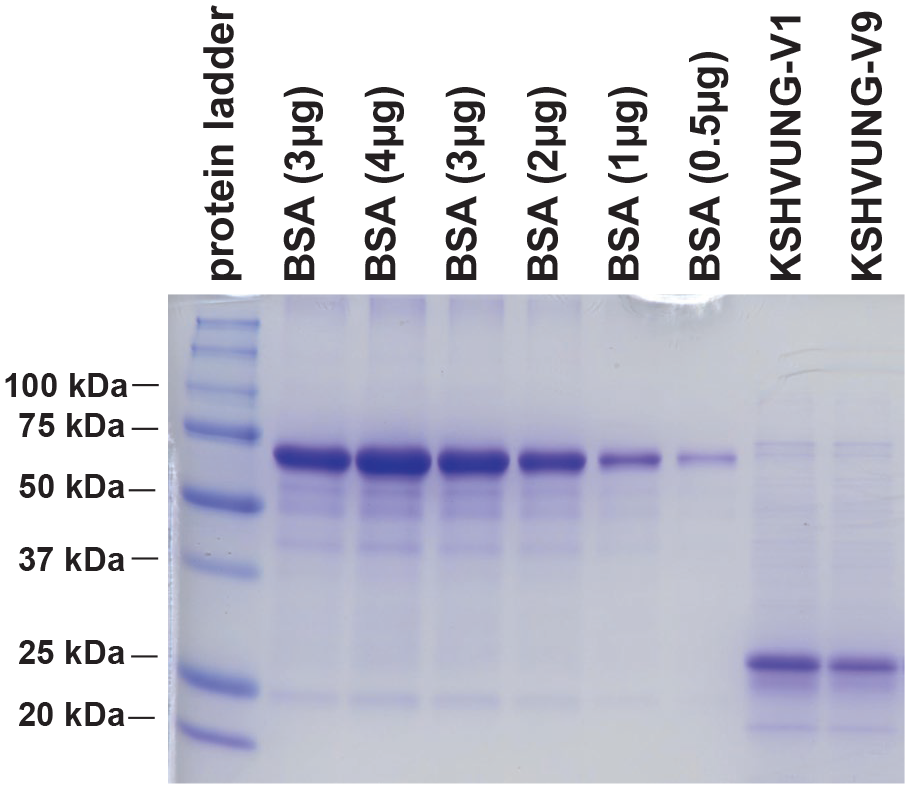

Supplement: S5 Fig — Recombinant UNGs were resolved by SDS-PAGE and stained with Coomassie brilliant blue R-250. Pre-stained protein ladder and serially diluted BSA standard were used for protein size estimation and quantification of recombinant KSHV UNGs. (TIF) [file ppat.1012338.s005.tif]
