# Supplementary material for: Sequencing of Kaposi’s Sarcoma Herpesvirus (KSHV) genomes from persons of diverse ethnicities and provenances with KSHV-associated diseases demonstrate multiple infections, novel polymorphisms, and low intra-host variance
Source: PLoS Pathog. 2024 Jul 15;20(7):e1012338. doi: 10.1371/journal.ppat.1012338 (PMC11271956; doi:10.1371/journal.ppat.1012338)
Supplement: S1 Table — Samples used in phylogenetic and PCA analyses are indicated in individual columns. Those sequences for which consensus sequences were submitted to GenBank and for whom short read data has been made available in SRA are also specified. (DOCX) [file ppat.1012338.s006.docx]

| **KSHV Genome** | **ID** | **Material** | **Date of Sample** | **KSHV K1 Subtype** | **KSHV K15 Subtype** | **Comment** | **GenBank Submission** | **GenBank/SRA Accession** | **K1 Gene Analysis (Figure 1A)** | **Whole Genome Analysis (Figure 1B)** | **PCA Analysis (Figure 2)** |
| --- | --- | --- | --- | --- | --- | --- | --- | --- | --- | --- | --- |
| 1 | FNL013_CA | Effusion | 1/28/2011 | A4 | P |  | 1 | OR829403 | X | X |  |
| 2 | FNL0015_NA | PBMC | 2/20/2018 | C3 | P |  | 2 | OR829339 | X | X | X |
| 3 | FNL0016_CA | PBMC | 2/19/2020 | A4 | P |  | 3 | OR829340 | X | X | X |
| 4 | FNL0018_NA | Lymph node | 7/9/2015 | A3 | P |  | 4 | OR829342 | X | X | X |
| 5 | FNL0019_NA | Effusion | 6/5/2013 | F2 and A3 | M | Mixed infection |  |  |  |  |  |
| 6 | FNL0024_NA | Oral Fluid | 4/15/2015 | A1 | P |  | 5 | OR829347 | X | X |  |
| 7 | FNL0025_NA | PBMC | 7/24/2013 | C1, C2, B1 and C1 | P and M | Mixed infection |  |  |  |  |  |
| 8 | FNL0026_WAF | PBMC | 10/28/2019 | B | M |  | 6 | OR829348 | X | X | X |
| 9 | FNL0027_SA | PBMC | 10/22/2014 | A4 | P |  | 7 | OR829349 | X | X |  |
| 10 | FNL0028_WAF | Biopsy | 2/27/2014 | B | P |  | 8 | OR829350 | X | X | X |
| 11 | FNL0029_NA | Oral Fluid | 7/11/2018 | B3 | M |  | 9 | OR829351 | X | X | X |
| 12 | FNL0030_WAF | PBMC | 9/18/2014 | A5 | P |  | 10 | OR829352 | X | X | X |
| 13 | FNL0031_WA | Oral Fluid | 6/8/2016 | A2 | P |  | 11 | OR829353 | X | X | X |
| 14 | FNL0033_NA | Oral Fluid | 2/2/2021 | A4 | P |  | 12 | OR829355 | X | X |  |
| 15 | FNL0036_NA | Oral Fluid | 12/11/2019 | B1 | M |  | 13 | OR829358 | X | X | X |
| 16 | FNL0037_NA | Oral Fluid | 5/25/2005 | C3 | P |  | 14 | OR829359 | X | X | X |
| 17 | FNL0038_CAR | Oral Fluid | 4/14/2005 | A1 | M |  | 15 | OR829360 | X | X | X |
| 18 | FNL0040_AMR | PBMC | 5/8/2019 | C3 | P |  | 16 | OR829362 | X | X |  |
| 19 | FNL0041_NA | Oral Fluid | 11/28/2018 | B3 and A4 | P and M | Mixed infection |  |  |  |  |  |
| 20 | FNL0042_NA | Effusion | 6/11/2018 | A4 | P |  | 17 | OR829363 | X | X |  |
| 21 | FNL0043_NA | PBMC | 12/13/2017 | C3 and B1 | P and M | Mixed infection |  |  |  |  |  |
| 22 | FNL0044_NA | Effusion | 3/11/2020 | A4 | P |  | 18 | OR829364 | X | X |  |
| 23 | FNL0046_NA | Oral Fluid | 10/25/2017 | C3 | M |  | 19 | OR829366 | X | X | X |
| 24 | FNL0049_SEU | Oral Fluid | 9/25/2013 | A4 | P |  | 20 | OR829369 | X | X |  |
| 25 | FNL0051_NA | Oral Fluid | 1/5/2021 | A4 | P |  | 21 | OR829371 | X | X |  |
| 26 | FNL0052_CA | PBMC | 10/16/2019 | B1 | M |  | 22 | OR829372 | X | X | X |
| 27 | FNL0053_NA | PBMC | 1/15/2020 | B1 | M |  | 23 | OR829373 | X | X | X |
| 28 | FNL0054_NA | Oral Fluid | 7/6/2016 | A3 | P |  | 24 | OR829374 | X | X | X |
| 29 | FNL0055_NA | Oral Fluid | 8/9/2017 | C2 | P |  | 25 | OR829375 | X | X | X |
| 30 | FNL0056_NA | PBMC | 6/28/2021 | A5 | M |  | 26 | OR829376 | X | X | X |
| 31 | FNL0060_EAF | Oral Fluid | 12/30/2020 | F | P |  | 27 | OR829377 | X | X | X |
| 32 | FNL0061_NA | PBMC | 4/28/2021 | A4 and B4 | P | Mixed infection |  |  |  |  |  |
| 33 | FNL0064_MAF | PBMC | 5/19/2021 | A | P |  | 28 | OR829380 | X | X | X |
| 34 | FNL0065_SEU | Oral Fluid | 4/29/2015 | C2 | P |  | 29 | OR829381 | X | X | X |
| 35 | FNL0066_CA | PBMC | 5/19/2021 | A4 | P |  | 30 | OR829382 | X | X |  |
| 36 | FNL0068_SA | PBMC | 8/19/2020 | C2 | P |  | 31 | OR829383 | X | X | X |
| 37 | FNL0070_CAR | Oral Fluid | 2/18/2015 | C3 | P |  | 32 | OR829385 | X | X |  |
| 38 | FNL0072_NA | Oral Fluid | 3/20/2019 | C7 | P |  | 33 | OR829387 | X | X |  |
| 39 | FNL0073_NA | PBMC | 2/14/2018 | B3 | M |  | 34 | OR829388 | X | X |  |
| 40 | FNL0076 | Biopsy | 12/10/2012 | C3 | P |  | 35 | OR829390 | X | X | X |
| 41 | FNL0077_NA | PBMC | 5/2/2018 | A2 | P |  | 36 | OR829391 | X | X | X |
| 42 | FNL0079_NA | Oral Fluid | 2/3/2016 | A4 | P |  | 37 | OR829393 | X | X |  |
| 43 | FNL0080_EA | Oral Fluid | 1/2/2019 | C2 | P |  | 38 | OR829394 | X | X | X |
| 44 | FNL0083_NA | PBMC | 9/4/2019 | A3 | P |  | 39 | OR829396 | X | X | X |
| 45 | FNL0084_NA | PBMC | 6/21/2007 | B1 | M |  | 40 | OR829397 | X | X | X |
| 46 | FNL0085_WAF | Oral Fluid | 4/16/2008 | B3 | P |  | 41 | OR829398 | X | X | X |
| 47 | FNL0086_NA | PBMC | 12/1/2017 | B1 | M |  | 42 | OR829399 | X | X | X |
| 48 | FNL0088_NA | Oral Fluid | 11/5/2014 | A4 | P |  | 43 | OR829400 | X | X |  |
| 49 | FNL0089_NA | Effusion | 1/6/2021 | A2 | P |  | 44 | OR829401 | X | X | X |
| 50 | FNL002_NA | PBMC | 11/6/2013 | A4 | P | 99.25% identical sites between sequences | 45 | OR829341 | X | X |  |
|  | FNL002_NA | Effusion | 3/25/2013 | A2 | P |  |  | MN419219.1 |  | X |  |
| 51 | FNL003_NA | PBMC | 1/3/2013 | A4 | P | 100% identical sites between sequences |  |  |  | X |  |
|  | FNL003_NA | Effusion | 5/30/2013 | A4 | P |  |  | MN419220.1 | X | X |  |
| 52 | FNL008_NA | PBMC | 12/12/2012 | B1 and A2 | M and P | Mixed infection in both |  |  |  |  |  |
| 53 | FNL008_NA | Effusion | 2/6/2013 | B1 and A2 | M and P |  |  |  |  |  |  |
| 54 | FNL010_NA | PBMC | 10/22/2014 | C3 | P | 100% identical sites between sequences | 46 | OR829402 | X | X | X |
| 55 | FNL010_NA | Effusion | 10/23/2014 | C3 | P |  |  |  |  | X |  |
| 56 | FNL0020_NA | PBMC | 3/4/2021 | A4 | P | 99.98% identical sites between sequences |  |  |  | X |  |
| 57 | FNL0020_NA | Effusion | 3/2/2021 | A4 | P |  | 47 | OR829343 | X | X |  |
| 58 | FNL0021_20050323_NA | PBMC | 3/23/2005 | C | P | Different KSHV subtype than later dates | 48 | OR829404 | X | X |  |
| 59 | FNL0021_NA | PBMC | 8/19/2005 | F2 | M | 100% identical sites between sequences |  |  |  | X |  |
| 60 | FNL0021_NA | PBMC | 3/15/2006 | F2 | M |  | 49 | OR829344 | X | X | X |
| 61 | FNL0022_NA | PBMC | 3/14/2018 | A3 | M | 100% identical sites between sequences | 50 | OR829345 |  | X | X |
| 62 | FNL0022_NA | Oral Fluid | 5/23/2018 | A3 | M |  |  |  |  | X |  |
| 63 | FNL0023_NA | Whole Blood | 6/30/2015 | A1 | P | 99.99% identical sites between sequences | 51 | OR829346 | X | X | X |
| 64 | FNL0023_NA | Biopsy | 7/31/2014 | A1 | P |  |  |  |  | X |  |
| 65 | FNL0032_NA | PBMC | 6/27/2012 | A1 | P | 99.99% identical sites between sequences |  |  |  | X |  |
| 66 | FNL0032_NA | Effusion | 1/26/2012 | A1 | P |  | 52 | OR829354 | X | X | X |
| 67 | FNL0034_NA | PBMC | 10/10/2019 | C3 | P | 99.98% identical sites between sequences |  |  |  | X |  |
| 68 | FNL0034_NA | Effusion | 10/23/2019 | C3 | P |  | 53 | OR829356 | X | X | X |
| 69 | FNL0035_NA | PBMC | 8/21/2014 | A4 | P | 99.97% identical sites between sequences |  |  |  | X |  |
| 70 | FNL0035_NA | Biopsy | 8/21/2014 | A4 | P |  | 54 | OR829357 | X | X |  |
| 71 | FNL0039_EAF | PBMC | 10/2602020 | B4 and A4 | M and P | Mixed infection in PBMC |  |  |  |  |  |
| 72 | FNL0039_EAF | Oral Fluid | 2/10/2021 | B4 | M |  | 55 | OR829361 | X | X | X |
| 73 | FNL0045_NA | PBMC | 9/22/2020 | C7 | P | 99.99% identical sites between sequences |  |  |  | X |  |
| 74 | FNL0045_NA | Effusion | 1/29/2020 | C7 | P |  | 56 | OR829365 | X | X | X |
| 75 | FNL0047_WAF | PBMC | 2/3/2020 | B3 | P | 99.91% identical sites between sequences |  |  |  | X |  |
| 76 | FNL0047_WAF | Effusion | 1/21/2020 | B3 | P |  | 57 | OR829367 | X | X | X |
| 77 | FNL0048_WAF | PBMC | 8/13/2017 | B3 | P | 99.99% identical sites between sequences |  |  |  | X |  |
| 78 | FNL0048_WAF | Effusion | 12/16/16 | B3 | P |  | 58 | OR829368 | X | X | X |
| 79 | FNL0050_NA | PBMC | 1/5/2021 | A4 | P | 100% identical sites between sequences |  |  | X | X |  |
| 80 | FNL0050_NA | Effusion | 1/5/2021 | A4 | P |  | 59 | OR829370 | X | X |  |
| 81 | FNL0059_NA | Bronchoalveolar lavage | 8/10/2020 | B1 and C7 | P | Mixed infection in both |  |  |  |  |  |
| 82 | FNL0059_NA | PBMC | 7/30/2020 | B1 and C7 | P |  |  |  |  |  |  |
| 83 | FNL0062_SA | Effusion | 8/6/2018 | E2 | P | 100% identical sites between sequences |  |  |  | X |  |
| 84 | FNL0062_SA | Oral Fluid | 8/6/2018 | E2 | P |  | 60 | OR829378 | X | X | X |
| 85 | FNL0063_EAF | PBMC | 6/4/2020 | B4 | P | 100% identical sites between sequences |  |  |  | X |  |
| 86 | FNL0063_EAF | Effusion | 6/3/2020 | B4 | P |  | 61 | OR829379 | X | X | X |
| 87 | FNL0067_CA | PBMC | 9/4/2019 | A5 and C3 | P and M | Mixed infection in both |  |  |  |  |  |
| 88 | FNL0067_CA | Oral Fluid | 4/10/2019 | A5 and C3 | P and M |  |  |  |  |  |  |
| 89 | FNL0069_NA | PBMC | 3/10/2021 | A | P | 99.99% identical sites between sequences |  |  |  | X |  |
| 90 | FNL0069_NA | Effusion | 4/19/2018 | A | P |  | 62 | OR829384 | X | X | X |
| 91 | FNL0071_SEU | Biopsy | 12/1/2012 | C2 | P |  | 63 | OR829386 | X | X |  |
| 92 | FNL0071_SEU | Oral Fluid | 3/28/2012 | C1 and C2 | P | Mixed infection in Oral Fluid |  |  |  |  |  |
| 93 | FNL0074_SA | PBMC | 11/6/2013 | A4 | P | 100% identical sites between sequences |  |  |  | X |  |
| 94 | FNL0074_SA | Biopsy | 12/19/2013 | A4 | P |  | 64 | OR829389 | X | X |  |
| 95 | FNL0078_NA | PBMC | 9/4/2019 | C3 | P | 100% identical sites between sequences | 65 | OR829392 | X | X | X |
| 96 | FNL0078_NA | Effusion | 9/4/2019 | C3 | P |  |  |  |  | X |  |
| 97 | FNL0081_NA | PBMC | 10/23/2019 | A3 | M | 100% identical sites between sequences |  |  |  | X |  |
| 98 | FNL0081_NA | Effusion | 1/2/2020 | A3 | M |  | 66 | OR829395 | X | X | X |
| 99 | FNL0082_NA | PBMC | 7/1/2020 | A4 and B1 | P | Mixed infection in both |  |  |  |  |  |
| 100 | FNL0082_NA | Oral Fluid | 6/23/2021 | A4 and B1 | P |  |  |  |  |  |  |
| 101 | FNL0090_NA | PBMC | 4/1/2019 | A4, C3, and B1 | P | Mixed infection in both |  |  |  |  |  |
| 102 | FNL0090_NA | PBMC | 4/24/2019 | A4 and B1 | P |  |  |  |  |  |  |
