# Supplementary material for: Sequencing of Kaposi’s Sarcoma Herpesvirus (KSHV) genomes from persons of diverse ethnicities and provenances with KSHV-associated diseases demonstrate multiple infections, novel polymorphisms, and low intra-host variance
Source: PLoS Pathog. 2024 Jul 15;20(7):e1012338. doi: 10.1371/journal.ppat.1012338 (PMC11271956; doi:10.1371/journal.ppat.1012338)
Supplement: S2 Table — (DOCX) [file ppat.1012338.s007.docx]

| **ID** | **raw** | **mapped** | **gk18_bases_cov** | **mean_cov** | **median_cov** | **cov_range** | **%>10X** | **Comment** |
| --- | --- | --- | --- | --- | --- | --- | --- | --- |
| FNL002_PBMC_S3_L001_R1_001_GK18 | 1591888 | 106429 | 137018 | 142.925 | 144 | 0-313 | 0.996103 |  |
| FNL003_PBMC_S3_L001_R1_001_GK18 | 2902028 | 157254 | 137405 | 199.477 | 206 | 0-296 | 0.991445 |  |
| FNL008_PBMC_S2_L001_R1_001_GK18 | 2277382 | 153961 | 136585 | 196.635 | 205 | 0-297 | 0.989627 |  |
| FNL008_PBMC_S2_L001_R1_001_U75698 | 2276544 | 154996 | 136913 | 197.674 | 204 | 0-308 | 0.993436 |  |
| FNL008_EFF_S2_L001_R1_001_GK18 | 2317458 | 140584 | 137426 | 196.024 | 196 | 0-462 | 0.994267 |  |
| FNL008_EFF_S2_L001_R1_001_U75698 | 2317918 | 140480 | 136823 | 197.851 | 197 | 0-495 | 0.996423 |  |
| FNL0015_PBMC_S3_L001_R1_001_GK18 | 4277400 | 124703 | 137283 | 177.051 | 181 | 0-258 | 0.993516 |  |
| FNL0016_PBMC_S1_L001_R1_001_GK18 | 3952670 | 144818 | 137393 | 193.194 | 202 | 0-294 | 0.993788 |  |
| FNL0017_PBMC_S1_L001_R1_001_GK18 | 21894 | 116 | 11259 | 1.5518 | 1 | 0-6 | 0 | Insufficient Coverage |
| FNL0018_LN_S2_L001_R1_001_GK18 | 1248156 | 126926 | 137861 | 178.122 | 181 | 0-291 | 0.994599 |  |
| FNL0019_EFF_S2_L001_R1_001_GK18 | 2662968 | 155587 | 135621 | 207.279 | 213 | 0-421 | 0.993392 |  |
| FNL0019_EFF_S2_L001_R1_001_U75698 | 2687878 | 157601 | 137012 | 208.103 | 213 | 0-382 | 0.994006 |  |
| FNL0020_PBMC_S1_L001_R1_001_GK18 | 1624884 | 143700 | 137551 | 182.396 | 186 | 0-308 | 0.996077 |  |
| FNL0020_EFF_S1_L001_R1_001_GK18 | 229934 | 144970 | 137535 | 189.388 | 194 | 0-291 | 0.996881 |  |
| FNL0021_031506_S1_L001_R1_001_GK18 | 300910 | 137660 | 135592 | 191.514 | 197 | 0-307 | 0.995229 |  |
| FNL0021_031506_S1_L001_R1_001_U75698 | 300218 | 138189 | 137057 | 190.02 | 194 | 0-279 | 0.995152 | K15 M allele |
| FNL0021_20050819_S1_L001_R1_001_GK18 | 1429434 | 133446 | 135387 | 181.186 | 193 | 0-299 | 0.989887 |  |
| FNL0021_20050819_S1_L001_R1_001_U75698 | 1430016 | 136178 | 136902 | 182.89 | 193 | 0-284 | 0.990527 | K15 M allele |
| FNL0021_PBMC_20050323_S2_L001_R1_001_GK18 | 6256610 | 144149 | 137296 | 187.479 | 201 | 0-335 | 0.993119 |  |
| FNL0022_PBMC_S1_L001_R1_001_GK18 | 557956 | 139985 | 135335 | 191.421 | 197 | 0-280 | 0.99454 |  |
| FNL0022_PBMC_S1_L001_R1_001_U75698 | 591328 | 142496 | 136829 | 192.889 | 197 | 0-367 | 0.994327 | K15 M allele |
| FNL0022_OF_S3_L001_R1_001_GK18 | 1135026 | 134450 | 135514 | 182.964 | 190 | 0-358 | 0.978084 |  |
| FNL0022_OF_S3_L001_R1_001_U75698 | 1135076 | 136165 | 136887 | 187.034 | 191 | 0-358 | 0.995084 | K15 M allele |
| FNL0023_WB_S3_L001_R1_001_GK18 | 3468382 | 116554 | 137215 | 157.736 | 163 | 0-279 | 0.99186 |  |
| FNL0023_BIO_S3_L001_R1_001_GK18 | 1165568 | 84187 | 137449 | 104.67 | 92 | 0-316 | 0.986507 |  |
| FNL0024_OF_S1_L001_R1_001_GK18 | 3258626 | 157910 | 137551 | 201.443 | 206 | 0-316 | 0.99572 |  |
|  |  |  |  |  |  |  |  |  |
| FNL0025_PBMC_S1_L001_R1_001_GK18 | 944056 | 132580 | 137319 | 182.939 | 184 | 0-522 | 0.995733 |  |
| FNL0026_PBMC_S2_L001_R1_001_GK18 | 328612 | 140754 | 135442 | 189.8 | 195 | 0-292 | 0.994062 |  |
| FNL0026_PBMC_S2_L001_R1_001_U75698 | 328424 | 142799 | 136724 | 191.559 | 196 | 0-331 | 0.99629 | K15 M allele |
| FNL0027_PBMC_S2_L001_R1_001_GK18 | 1382990 | 137856 | 137482 | 187.509 | 192 | 0-293 | 0.996152 |  |
| FNL0028_BIO_S3_L001_R1_001_GK18 | 972858 | 136374 | 137909 | 185.147 | 190 | 0-341 | 0.994455 |  |
| FNL0029_OF_S2_L001_R1_001_GK18 | 402258 | 152822 | 135397 | 194.243 | 200 | 0-312 | 0.993674 |  |
| FNL0029_OF_S2_L001_R1_001_U75698 | 401956 | 154733 | 136881 | 194.435 | 199 | 0-279 | 0.996192 | K15 M allele |
| FNL0030_PBMC_S3_L001_R1_001_GK18 | 384460 | 136092 | 137823 | 173.754 | 178 | 0-301 | 0.994575 |  |
| FNL0031_OF_S1_L001_R1_001_GK18 | 1542782 | 144800 | 137249 | 191.682 | 196 | 0-444 | 0.996231 |  |
| FNL0032_PBMC_S2_L001_R1_001_GK18 | 3879986 | 67914 | 137075 | 92.2464 | 90 | 0-254 | 0.985164 |  |
| FNL0032_EFF_S3_L001_R1_001_GK18 | 3124360 | 151782 | 137844 | 202.281 | 208 | 0-329 | 0.994139 |  |
| FNL0033_OF_S2_L001_R1_001_GK18 | 1645412 | 139136 | 137603 | 186.881 | 192 | 0-271 | 0.995088 |  |
| FNL0034_PBMC_S2_L001_R1_001_GK18 | 1329934 | 49478 | 136425 | 66.3879 | 65 | 0-241 | 0.979247 |  |
| FNL0034_EFF_S1_L001_R1_001_GK18 | 267626 | 122760 | 137375 | 176.994 | 181 | 0-281 | 0.998079 |  |
| FNL0035_PBMC_S1_L001_R1_001_GK18 | 2337606 | 150659 | 137430 | 201.573 | 206 | 0-308 | 0.996415 |  |
| FNL0035_BIO_S2_L001_R1_001_GK18 | 1867162 | 139237 | 137730 | 187.535 | 192 | 0-305 | 0.993787 |  |
| FNL0036_OF_S3_L001_R1_001_GK18 | 1010194 | 145596 | 135293 | 194.094 | 200 | 0-307 | 0.994558 |  |
| FNL0036_OF_S3_L001_R1_001_U75698 | 1284968 | 145025 | 136978 | 191.765 | 197 | 0-276 | 0.994829 | K15 M allele |
| FNL0037_OF_S2_L001_R1_001_GK18 | 2030282 | 78968 | 137065 | 99.8686 | 96 | 0-311 | 0.993194 |  |
| FNL0038_OF_S1_L001_R1_001_GK18 | 2620058 | 141252 | 135527 | 179.52 | 188 | 0-310 | 0.974609 |  |
| FNL0038_OF_S1_L001_R1_001_U75698 | 2619294 | 143067 | 136892 | 183.334 | 188 | 0-293 | 0.99324 | K15 M allele |
| FNL0039_PBMC_S3_L001_R1_001_GK18 | 261776 | 133126 | 137873 | 179.558 | 186 | 0-335 | 0.992765 |  |
| FNL0039_PBMC_S3_L001_R1_001_U75698 | 261736 | 134591 | 137043 | 182.51 | 187 | 0-348 | 0.993867 | K15 M allele |
| FNL0039_OF_S2_L001_R1_001_GK18 | 1200264 | 148129 | 135516 | 184.659 | 192 | 0-398 | 0.978515 |  |
| FNL0039_OF_S2_L001_R1_001_U75698 | 1290544 | 148957 | 137031 | 186.867 | 191 | 0-369 | 0.995733 | K15 M allele |
| FNL0040_PBMC_S2_L001_R1_001_GK18 | 1093104 | 142718 | 137476 | 190.821 | 195 | 0-287 | 0.996366 |  |
| FNL0040_PBMC_S3_L001_R1_001_GK18 | 1385018 | 142381 | 137347 | 185.628 | 190 | 0-296 | 0.997736 |  |
| FNL0041_PBMC_S3_L001_R1_001_GK18 | 4362138 | 9278 | 85947 | 20.1676 | 18 | 0-112 | 0.666107 |  |
| FNL0041_PBMC_S3_L001_R1_001_U75698 | 2891584 | 6454 | 93801 | 12.902 | 11 | 0-78 | 0.56991 | K15 M allele |
| FNL0041_OF_S2_L001_R1_001_GK18 | 38284 | 35849 | 133801 | 38.7392 | 38 | 0-113 | 0.96003 |  |
| FNL0041_OF_S2_L001_R1_001_U75698 | 36148 | 35956 | 134739 | 38.609 | 38 | 0-113 | 0.941776 | K15 M allele |
| FNL0041_OF_S3_L001_R1_001_GK18 | 222322 | 156347 | 136041 | 193.68 | 202 | 0-306 | 0.978311 |  |
| FNL0041_OF_S3_L001_R1_001_U75698 | 222652 | 159070 | 136884 | 198.483 | 203 | 0-301 | 0.996112 | K15 M allele |
| FNL0042_EFF_S1_L001_R1_001_GK18 | 597608 | 148216 | 137558 | 190.419 | 195 | 0-306 | 0.995827 |  |
| FNL0043_PBMC_S3_L001_R1_001_GK18 | 2789694 | 160798 | 137370 | 205.855 | 198 | 0-456 | 0.991577 |  |
| FNL0044_EFF_S1_L001_R1_001_GK18 | 595592 | 129199 | 137402 | 178.489 | 183 | 0-264 | 0.994432 |  |
| FNL0045_PBMC_S1_L001_R1_001_GK18 | 3218530 | 142808 | 137446 | 190.88 | 202 | 0-301 | 0.991976 |  |
| FNL0045_EFF_S1_L001_R1_001_GK18 | 2259878 | 162522 | 137525 | 205.054 | 212 | 0-307 | 0.995674 |  |
| FNL0046_OF_S1_L001_R1_001_GK18 | 270928 | 55371 | 134554 | 71.572 | 70 | 0-257 | 0.982498 |  |
| FNL0046_OF_S1_L001_R1_001_U75698 | 271372 | 56033 | 136114 | 71.9102 | 70 | 0-207 | 0.98644 | K15 M allele |
| FNL0047_PBMC_S1_L001_R1_001_GK18 | 3531646 | 61696 | 136831 | 79.1509 | 75 | 0-255 | 0.979077 |  |
| FNL0047_EFF_S3_L001_R1_001_GK18 | 2251004 | 151006 | 137547 | 199.761 | 208 | 0-310 | 0.990503 |  |
| FNL0048_PBMC_S1_L001_R1_001_GK18 | 1083698 | 145004 | 137421 | 178.423 | 183 | 0-291 | 0.993895 |  |
| FNL0048_EFF_S2_L001_R1_001_GK18 | 2008044 | 158882 | 137877 | 201.392 | 209 | 0-315 | 0.99188 |  |
| FNL0049_OF_S3_L001_R1_001_GK18 | 2130364 | 153188 | 137470 | 195.48 | 199 | 0-305 | 0.9968 |  |
| FNL0050_PBMC_S3_L001_R1_001_GK18 | 2064590 | 133680 | 137535 | 174.844 | 179 | 0-278 | 0.994929 |  |
| FNL0050_EFF_S2_L001_R1_001_GK18 | 1629936 | 129905 | 137225 | 168.665 | 173 | 0-308 | 0.995239 |  |
| FLN0051_OF_S1_L001_R1_001_GK18 | 2473290 | 136524 | 137616 | 182.755 | 187 | 0-272 | 0.996258 |  |
| FNL0052_PBMC_S1_L001_R1_001_GK18 | 1299822 | 134598 | 135213 | 185.95 | 191 | 0-314 | 0.992598 |  |
| FNL0052_PBMC_S1_L001_R1_001_U75698 | 1300518 | 137451 | 136839 | 188.192 | 193 | 0-312 | 0.991857 | K15 M allele |
| FNL0053_PBMC_S3_L001_R1_001_GK18 | 3494670 | 68548 | 134815 | 94.1424 | 91 | 0-267 | 0.982884 |  |
| FNL0053_PBMC_S3_L001_R1_001_U75698 | 3758974 | 69307 | 136453 | 94.1822 | 92 | 0-257 | 0.98638 |  |
| FNL0054_OF_S1_L001_R1_001_GK18 | 1344724 | 140530 | 137579 | 173.533 | 178 | 0-264 | 0.991136 |  |
| FNL0055_OF_S2_L001_R1_001_GK18 | 698640 | 141985 | 137582 | 177.878 | 182 | 0-307 | 0.995109 |  |
| FNL0056_PBMC_S2_L001_R1_001_GK18 | 268808 | 138922 | 135544 | 190.128 | 195 | 0-381 | 0.993957 |  |
| FNL0056_PBMC_S2_L001_R1_001_U75698 | 268626 | 140286 | 137047 | 190.912 | 195 | 0-303 | 0.994472 | K15 M allele |
| FNL0059_BAL_S2_L001_R1_001_GK18 | 100124 | 76162 | 135858 | 97.0011 | 93 | 0-312 | 0.986114 |  |
| FNL0059_PBMC_S2_L001_R1_001_GK18 | 2403316 | 162706 | 137217 | 212.271 | 215 | 0-487 | 0.994229 |  |
| FNL0060_OF_S1_L001_R1_001_GK18 | 2763282 | 158318 | 137425 | 200.922 | 207 | 0-306 | 0.993358 |  |
| FNL0061_PBMC_S3_L001_R1_001_GK18 | 810546 | 150211 | 137218 | 193.134 | 196 | 1-475 | 0.996932 |  |
| FNL010_PBMC_S3_L001_R1_001_GK18 | 1901392 | 155261 | 137184 | 202.027 | 207 | 0-307 | 0.996572 |  |
| FNL010_EFF_S2_L001_R1_001_GK18 | 1905450 | 151927 | 137299 | 201.113 | 207 | 0-289 | 0.995011 |  |
| FNL0062_EFF_S1_L001_R1_001_GK18 | 3250924 | 153820 | 137318 | 202.909 | 209 | 0-330 | 0.991307 |  |
| FNL0062_OF_S3_L001_R1_001_GK18 | 1891596 | 155852 | 137314 | 188.265 | 194 | 0-305 | 0.990654 |  |
| FNL0063_PBMC_S2_L001_R1_001_GK18 | 2284156 | 152049 | 137537 | 196.127 | 201 | 0-322 | 0.996053 |  |
| FNL0063_EFF_S1_L001_R1_001_GK18 | 309298 | 148286 | 137720 | 194.124 | 199 | 0-361 | 0.99323 |  |
| FNL0064_PBMC_S3_L001_R1_001_GK18 | 2070586 | 143955 | 137486 | 191.134 | 198 | 0-380 | 0.992956 |  |
| FNL0065_OF_S3_L001_R1_001_GK18 | 2121470 | 138573 | 137386 | 186.808 | 192 | 0-311 | 0.993887 |  |
| FNL013_EFF_S1_L001_R1_001_GK18 | 1445556 | 130950 | 137485 | 178.51 | 182 | 0-273 | 0.997041 |  |
| FNL013_EFF_S1_L001_R1_001_U75698 | 1445618 | 128274 | 134582 | 178.438 | 182 | 0-285 | 0.995791 | K15 M allele |
| FNL0066_PBMC_S1_L001_R1_001_GK18 | 2809020 | 91865 | 137386 | 120.441 | 118 | 0-266 | 0.991695 |  |
| FNL0066_PBMC_S2_L001_R1_001_GK18 | 4422372 | 146532 | 137288 | 197.097 | 205 | 0-334 | 0.993309 |  |
| FNL0066_OF_41019_S1_L001_R1_001_GK18 | 239892 | 25567 | 134843 | 34.8533 | 34 | 0-283 | 0.923126 |  |
| FNL0068_PBMC_S2_L001_R1_001_GK18 | 621672 | 135847 | 137322 | 183.235 | 188 | 0-301 | 0.994721 |  |
| FNL0069_PBMC_S3_L001_R1_001_GK18 | 2802810 | 112008 | 136988 | 150.409 | 158 | 0-357 | 0.991583 |  |
| FNL0069_EFF_S2_L001_R1_001_GK18 | 1537564 | 153095 | 137419 | 199.061 | 204 | 0-299 | 0.996923 |  |
| FNL0070_OF_S3_L001_R1_001_GK18 | 2456122 | 150632 | 137295 | 198.111 | 204 | 0-294 | 0.995001 |  |
| FNL0071_BIO_S2_L001_R1_001_GK18 | 2951376 | 130597 | 137861 | 183.968 | 187 | 0-351 | 0.995289 |  |
| FNL0071_OF_S3_L001_R1_001_GK18 | 1319434 | 148721 | 137541 | 190.526 | 195 | 0-412 | 0.997296 |  |
| FNL0072_OF_S2_L001_R1_001_GK18 | 245322 | 57474 | 136774 | 76.288 | 75 | 0-262 | 0.988224 |  |
| FNL0073_PBMC_S3_L001_R1_001_GK18 | 571404 | 153540 | 135555 | 200.332 | 207 | 0-303 | 0.992002 |  |
| FNL0073_PBMC_S3_L001_R1_001_U75698 | 571898 | 156454 | 136933 | 202.849 | 208 | 0-310 | 0.996846 | K15 M allele |
| FNL0074_PBMC_S2_L001_R1_001_GK18 | 3130674 | 85213 | 137087 | 115.62 | 113 | 0-297 | 0.990513 |  |
| FNL0074_BIO_S3_L001_R1_001_GK18 | 1206190 | 119984 | 137631 | 170.3 | 174 | 0-313 | 0.994107 |  |
| FNL0076_BIO_S2_L001_R1_001_GK18 | 479870 | 132771 | 137624 | 172.07 | 175 | 0-302 | 0.994115 |  |
| FNL0077_PBMC_S2_L001_R1_001_GK18 | 1658738 | 140379 | 137279 | 191.154 | 196 | 0-295 | 0.993222 |  |
| FNL0078_PBMC_S3_L001_R1_001_GK18 | 3973064 | 146765 | 137207 | 194.615 | 203 | 0-299 | 0.993051 |  |
| FNL0078_EFF_S1_L001_R1_001_GK18 | 3614338 | 125315 | 137352 | 179.893 | 184 | 0-312 | 0.994053 |  |
| FNL0079_OF_S1_L001_R1_001_GK18 | 1041990 | 148506 | 137536 | 188.832 | 193 | 0-290 | 0.996881 |  |
| FNL0080_OF_S3_L001_R1_001_GK18 | 499094 | 154378 | 137252 | 188.605 | 194 | 0-294 | 0.995099 |  |
| FNL0081_PBMC_S2_L001_R1_001_GK18 | 2448834 | 141344 | 134565 | 179.705 | 192 | 0-292 | 0.993679 |  |
| FNL0081_PBMC_S2_L001_R1_001_U75698 | 2447992 | 143895 | 136435 | 180.52 | 193 | 0-302 | 0.993825 | K15 M allele |
| FNL0081_EFF_S2_L001_R1_001_GK18 | 2635604 | 143607 | 135313 | 195.196 | 204 | 0-332 | 0.980458 |  |
| FNL0081_EFF_S2_L001_R1_001_U75698 | 2657276 | 144327 | 136693 | 197.266 | 202 | 0-303 | 0.993889 |  |
| FNL0082_PBMC_S3_L001_R1_001_GK18 | 2326656 | 161097 | 137317 | 203.096 | 207 | 0-445 | 0.995643 |  |
| FNL0082_OF_S3_L001_R1_001_GK18 | 1083250 | 147038 | 137309 | 190.749 | 195 | 0-439 | 0.99589 |  |
| FNL0083_PBMC_S1_L001_R1_001_GK18 | 3454920 | 128759 | 137219 | 181.669 | 187 | 0-280 | 0.992248 |  |
| FNL0084_PBMC_S3_L001_R1_001_GK18 | 600024 | 132880 | 135510 | 188.328 | 194 | 0-292 | 0.992525 |  |
| FNL0084_PBMC_S3_L001_R1_001_U75698 | 601150 | 135194 | 136757 | 189.845 | 195 | 0-307 | 0.992891 |  |
| FNL0085_OF_S1_L001_R1_001_GK18 | 347224 | 115046 | 137111 | 161.194 | 172 | 0-287 | 0.992609 |  |
| FNL0086_PBMC_S1_L001_R1_001_GK18 | 2123272 | 153260 | 135208 | 200.986 | 207 | 0-295 | 0.991262 |  |
| FNL0086_PBMC_S1_L001_R1_001_U75698 | 2123054 | 155304 | 136949 | 201.816 | 207 | 0-321 | 0.994813 |  |
| FNL0088_OF_S2_L001_R1_001_GK18 | 2849752 | 147399 | 137583 | 191.766 | 196 | 0-329 | 0.994886 |  |
| FNL0089_EFF_S3_L001_R1_001_GK18 | 5454430 | 151611 | 137321 | 198.977 | 205 | 0-333 | 0.994464 |  |
| FNL0090_PBMC_24_S4_L001_R1_001_GK18 | 2775978 | 138189 | 137877 | 186.147 | 190 | 0-408 | 0.993134 |  |
| FNL0090_PBMC_S2_L001_R1_001_GK18 | 1606542 | 162149 | 137935 | 204.589 | 202 | 0-630 | 0.997499 |  |
| FNL0091_OF_S1_L001_R1_001_GK18 | 4423138 | 233 | 1871 | 13.8246 | 5 | 0-90 | 0.302757 | Insufficient Coverage |
