# Supplementary material for: Sequencing of Kaposi’s Sarcoma Herpesvirus (KSHV) genomes from persons of diverse ethnicities and provenances with KSHV-associated diseases demonstrate multiple infections, novel polymorphisms, and low intra-host variance
Source: PLoS Pathog. 2024 Jul 15;20(7):e1012338. doi: 10.1371/journal.ppat.1012338 (PMC11271956; doi:10.1371/journal.ppat.1012338)
Supplement: S3 Table — (DOCX) [file ppat.1012338.s008.docx]

| **Name** | **K1 Subtype** | **Primer** | **Product Size** |
| --- | --- | --- | --- |
|  |  |  |  |
| \| FNL0071 \| \| --- \| \|  \| | C1 | **AA**TTTGTGCCCTGGAGTGATTTCAA | 650 bp |
|  | C2 | **CC**TTTGTGCCCTGGAGTGATT | 650 bp |
|  | Reverse | CCTAAGATACCACACATGGTCC |  |
|  |  |  |  |
| \| FNL090 \| \| --- \| \|  \| | A4 | T**T**TCCGACTGACG**G**AG**AG**AACA | 550 bp |
|  | C3 | T**C**TCCGACTGACG**C**AG**GA**AA | 550 bp |
|  | B1 | **CA**CCGACTAACGGCG**TCT**AA | 549 bp |
|  | Reverse | GATACCACACACATGGTTCCTAT |  |
| \| FNL008 \| \| --- \| \|  \| | C3 | **Use FNL0090 Forward primer** | 730 bp |
|  | A2 | **AAT**ACTCGGCTTT**TG**CGACTGAC | 782 bp |
|  | B1 | **GGG**ACTCGGCTT**CA**CCGA | 782 bp |
|  | Reverse | **Use FNL0039 reverse primer** |  |
| FNL0061; FNL0082 | A4 | **G**A**GG**ACTATT**A**AGCCTT**TC**TCTG | 700 bp |
|  | B4 | **C**A**AA**ACTATT**G**AGCCTT**CA**TCTGC | 700 bp |
|  | Reverse | CCTAAGATACCACACATGGTTC |  |
|  |  |  |  |
| \| FNL0043; FNL0059 \| \| --- \| \|  \| | B1 | **G**TT**G**T**T**T**G**CA**G**T**C**TG**GC**GGTTT | 732 bp |
|  | C3 | **T**TT**C**T**C**T**A**CA**T**T**T**TG**CT**GGTTTGCTTT | 732 bp |
|  | Reverse | AGTTCCTAAGATACCACACATGGTT |  |
|  |  |  |  |
| FNL0019 | A3 | **AAT**ACTC**G**GCTT**TGG**CGACTG | 500 bp |
|  | F2 | **GGA**ACTC**A**GCTT**CTT**CGACTG | 500 bp |
|  | Reverse | GATTTTGCTTCTTTCACTACAACTCA |  |
|  |  |  |  |
| FNL0025 | B1 | **C**A**AA**ACTATT**G**AGCCTT**CA**TCTGC | 397 bp |
|  | C1 | **G**T**GG**ATCCTTGCCAACA**A**CGT | 362 bp |
|  | C3 | **CC**T**T**TGTGCCCTGGAGTGATT | 362 bp |
|  | C2 | **A**T**AC**ATCCTTGCCAACA**T**CCTGG | 310 bp |
|  | Reverse | GTAAAATTATAGTATTTAGTTTGTGACACGG |  |
| \| FNL0067 \| \| --- \| | A5 | **TGG**CGACTGA**G**G**G**A**CC**AA**T** | 558 bp |
|  | C3 | **CTC**CGACTGA**C**G**C**A**GG**AA**A** | 558 bp |
|  | Reverse | **Use FNL0059 revere primer** |  |
| FNL0039 | A4 | **AC**ATGG**C**AT**G**CACAACCTGT | 634 bp |
|  | B1 | **GA**ATGG**T**AT**A**CACAACCTGT | 634 bp |
|  | Reverse | CATTTGTAGCTATAAACATA |  |
| FNL0041 | A4 | ATACTCGGCTTTTCCGACTGAC | 665 bp |
|  | B3 | GGACTCGGCTTTGGCGAATA | 665 bp |
|  | Reverse | **Use FNL0061 reverse primer** |  |
