# Supplementary material for: Sequencing of Kaposi’s Sarcoma Herpesvirus (KSHV) genomes from persons of diverse ethnicities and provenances with KSHV-associated diseases demonstrate multiple infections, novel polymorphisms, and low intra-host variance
Source: PLoS Pathog. 2024 Jul 15;20(7):e1012338. doi: 10.1371/journal.ppat.1012338 (PMC11271956; doi:10.1371/journal.ppat.1012338)
Supplement: S1 Document — (DOCX) [file ppat.1012338.s010.docx]

S1 Document

De novo assembly pipeline

Raw paired-end fastq data are trimmed of standard Illumina adapter sequences and filtered for minimum length and quality scores using BBTools/bbduk.sh (1) with settings `ktrim=r k=21 mink=8 hdist=1 qtrim=rl trimq=28 minlen=50 tbo tpe. FastQC (2) reports are generated pre- and post-trimming to verify effective initial QC processing. Human, PHIX, EBV, and KSHV reads are individually isolated from trimmed data using BBTools/bbsplit.sh (Table S1). KSHV-specific reads are then optionally normalized to a maximum of 200X coverage with BBTools/bbnorm.sh (1).

The resulting trimmed and filtered reads are first mapped to NC_009333.1 (GK18) with BBTools/bbmap.sh to generate a reference-guided alignment (1). The resulting SAM file is converted to BAM with samtools -view (3), then passed to picard/CollectInsertSizeMetrics4 to determine mean paired-end insert size (4).

The same trimmed reads are then passed to the de novo assembly process. The genome structure of KSHV presents a distinct challenge in de novo assembly. To avoid introduction of algorithm-specific assembly artifacts, and to maximize chance of a successful assembly, reads are processed with three de bruijn graph-based methods: SPAdes –careful (5), SPAdes –meta (5), and MEGAHIT (6). Overlap layout consensus methods were considered, but ultimately discarded due to assembly time constraints. SPAdes --meta is specifically included to address potential multiple infections. Samples for which multiple K1/K15 subtypes are identified among contigs are flagged for further verification in a separate process described below.

The three resulting sets of assembled de novo contigs, the trimmed reads, and the picard-derived insert size metrics are subsequently passed to SSPACE (7) for contig extension, resulting in three sets of extended contigs. The initial and extended contig sets (six total assemblies) are then individually filtered for KSHV-specific contigs via BLAST (8) against a KSHV-specific blast database. The six final filtered contig sets are then each subjected to multiple-reference-guided scaffolding with MeDuSa (9), producing a single near-full-length scaffold per assembly. A multiple sequence alignment of the six draft genomes is performed with MAFFT (10) with --auto, --adjustdirection and --reorder settings (Figure S1.1). The alignment is then visually inspected and used to derive a final full-length consensus assembly using Geneious Prime (11), with a two thirds consensus set as the cutoff for final calls.


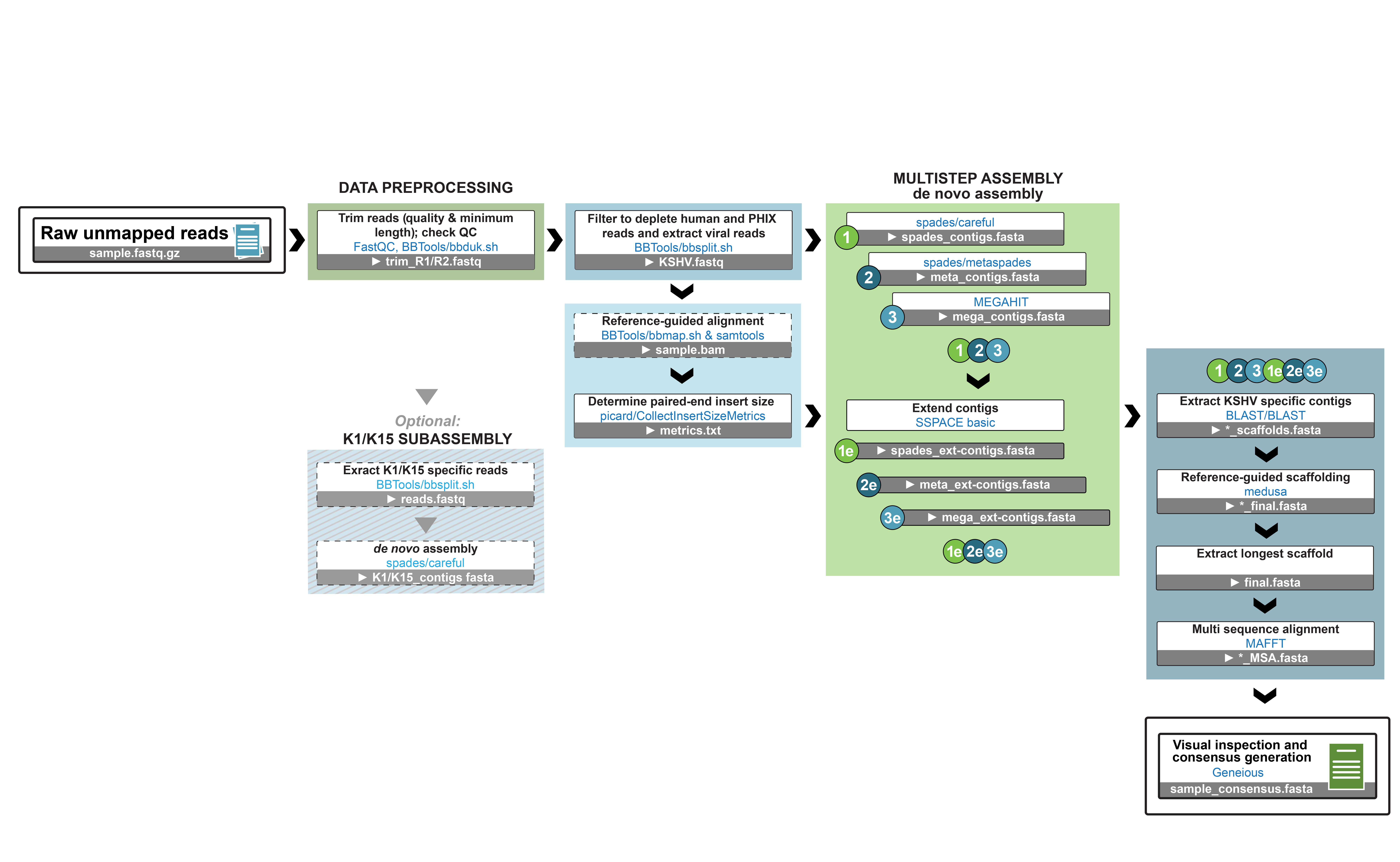


Figure S1.1: Schematic representation of the de novo assembly pipeline.

Optionally, de novo subassemblies of the K1 and K15 gene regions are performed. Trimmed reads are filtered against either a K1- or K15-specific database using BBTools/bbsplit.sh (1), and filtered reads are assembled de novo via SPAdes –careful (5). The advantage of this process is that this may be used to rapidly assess sample subtypes and assess putative multiple infection when full length assemblies are not required or possible.

Though the present study did not utilize unique molecular identifiers (UMIs), should they be added to the library preparation, UMIs are extracted from the raw fastq reads and appended to the read name using UMItools extract (12). Three base pairs (bp) are hard trimmed from the 5’ end of the read using BBTools/reformat.sh (1). Prior to deduplication, adapter sequences and UMIs (‘literal’) are removed from the 3’ end using BBTools/bbduk.sh and a BAM file generated from the reference-guided alignment against GK18 as described above. The processed reads are deduplicated using UMITools/dedup and exported to a fastq file using samtools -sort (3) and BBTools/bamtofastq. The UMI deduplicated reads are then processed through the pipeline as described above to generate sample-specific consensus scaffolds and K1/K15 subassemblies.

**Mixed infection verification and analysis**

In several samples, we obtained more than one complete assembly of the K1 region. In such cases, manually observed patterns of read mapping across the genome further suggested the possibility of mixed infection. Such patterns generally presented as a series of sequence variants which occurred in relatively consistent proportions at positions throughout the genome. Because error is a pervasive feature in next generation sequencing (13), and because the frequency of minor genome variants could approach the threshold of detection, we sought to develop a quantitative method for distinguishing signals of putative multiple infections from background sequencing error. Importantly, this process is conceptually distinct from haplotyping and variant calling methods. Rather than attempting to identify polymorphism, ours should be considered as a method for probabilistically identifying and filtering sequencing noise.

Data are initially filtered and assembled as described in the de novo assembly section. For each putative multiple-infected sample, a reference genome is defined as the consensus de novo assembly for the dominant genome variant. Trimmed reads are mapped onto the sample consensus as described for de novo assembly. The resulting BAM file is filtered for PCR duplicates detected by picard/MarkDuplicates (4). Reads present within the known internal repeat regions are excluded from the BAM using bedtools intersect -v2 (14). Using samtools mpileup (3), a per-position summary of coverage is then generated, omitting repeat regions (-l), disabling the base alignment quality computation (-B), with a maximum depth of 1000000 (-d), and setting the minimum base quality at 20 (-Q).

For each genome position, we have a fixed number of independent observations equal to the total coverage at that position. Each observation, the nucleotide reported per read, can be defined as either a success (reporting the expected nucleotide), or a failure (reporting any other nucleotide). The probability of “success” can be defined by an expected sequencing error/substitution rate. Meeting the necessary assumptions, we can model sequencing error as a binomial process:

$$\mathbf{P}\left( \boldsymbol{X=}\boldsymbol{k} \right)\boldsymbol{=}\binom{\boldsymbol{n}}{\boldsymbol{k}}\boldsymbol{p}^{\boldsymbol{k}}{\boldsymbol{(1}\boldsymbol{-p}\boldsymbol{)}}^{\boldsymbol{n-}\boldsymbol{k}}$$

in which the probability of a base call given the identity of the reference base $P\left( X=k \right)$ is determined by $n$ = the coverage at a given position, $p$ = the probability of base substitution, and $k$ = the total number “successes” or reported calls for the base in question. Conceptually, we’re determining whether a given base is observed to occur more frequently than would be expected due to random or systematic error$k=1$ C is observed in a position with coverage $n=1000,$ where the reference calls A, with a theoretical probability of randomly substitution is $p$ = 0.0002, the uncorrected probability of observing 1 or more Cs is p = 0.1813. This C would be flagged as noise, even prior to correction for multiple tests. A limitation of this method is that low-coverage sites may be prone to false-positive results. We find this to be acceptable given that the goal of this process is ultimately to visualize a broad cross-genome signal, rather than call individual site mutations, and positions with coverage below 20 are removed in subsequent steps prior to visualization.

Extensive consideration was given to the determination of appropriate substitution probabilities, or error rates, which we define as the number of non-reference base calls divided by the total coverage. Under the assumption that a reference cell line should harbor a non-significant number of actual polymorphisms, error rates were determined empirically by sequencing colonies from single-cell dilutions of reference cell line VG1 (15, 16) with each of three common library preparation protocols. These include Agilent SureSelect XT (XT, *exclusively utilized in the main study*), Roche KAPA/Universal Adapter (UA), and Roche KAPA/Universal UMI Adapter (UMI) protocols. Observed mapping frequencies are shown in table S1.1.

| *Table S1.1 Observed per-base mapping frequencies* | | | | |
| --- | --- | --- | --- | --- |
|  | Ref = A | Ref = C | Ref = G | Ref = T |
| *Agilent SureSelect XT* | | | | |
| A | 239875632 | 40521 | 62549 | 23308 |
| C | 9531 | 272848172 | 10966 | 41559 |
| G | 42986 | 11631 | 258600742 | 8896 |
| T | 24327 | 62704 | 31874 | 232211779 |
|  |  |  |  |  |
| *Roche KAPA Universal Adapter* | | | | |
| A | 9386333 | 2277 | 2927 | 985 |
| C | 410 | 10675227 | 630 | 1284 |
| G | 1400 | 690 | 10059698 | 392 |
| T | 1051 | 3173 | 2109 | 8916287 |
|  |  |  |  |  |
| *Roche KAPA UMI* | | | | |
| A | 86571579 | 24104 | 37419 | 13483 |
| C | 5795 | 99995308 | 5530 | 17746 |
| G | 18814 | 6175 | 94258639 | 5525 |
| T | 14335 | 38967 | 22680 | 82536742 |

To determine an appropriate substitution probability for use in the binomial test process, it was necessary to assess whether we observed significant differences in error rates which were either library-, base-, or substitution-dependent.

In comparing *total error rates per library*, we first visualized probability density functions of observed per-position error rates across VG1 (fig S1.2). Error rates are determined by mapping trimmed, deduplicated reads against the VG-1 reference genome. Per position, we calculate each base frequency divided by total coverage, resulting in 1 to 4 rates per position, depending on number of bases observed. Correct calls approach 100% at most positions and are omitted from the plot curve due to scale issues; these would appear as an extreme peak on the right side of the figure. We applied a Kruskal-Wallis test with Dunn’s *post hoc* test for multiple comparisons to the three complete distributions of empirically observed mapping rates, including all correct and errant base call rates, across the genome. We concluded that error rate profiles are distinct and library-dependent, thus precluding use of a simple unified value such as a total or mean error rate for our binomial probability. Note that substantially lower sequencing depth (~ 300X coverage) in the KAPA UA library likely contributed to the distinct peak observed at higher overall error proportion.


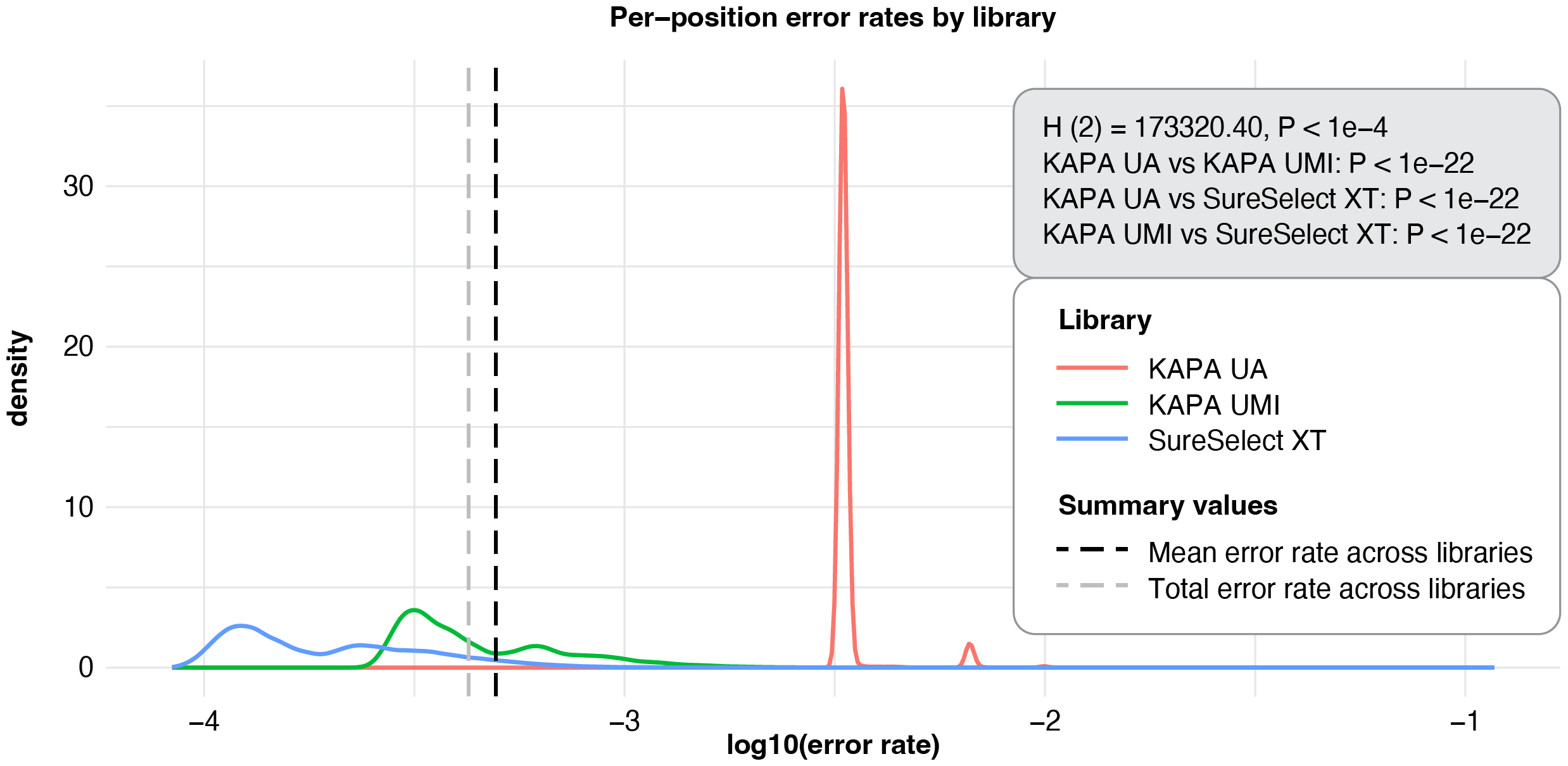


Figure S1.2, Probability density curves indicate the distribution of observed per-position error rates across VG-1 for each of three library protocols. Rates for correct calls were omitted from the density plot due to scale conflict. Curve color indicates library. Grey and black dashed vertical lines indicate mean and total error rates, respectively, across libraries. Kruskal-Wallis and Dunn’s test results comparing the complete distribution of per-position mapping rates are listed in the grey legend box.

We examined *total error rates per base* for each library in a similar manner. Plotting probability density functions for per-position error rates, binned by reference base, indicated a visible difference in each base’s error profile – though less drastic than what we observed at the library level. For each of the three libraries, a Kruskal-Wallis test confirmed significant difference in the four error profiles arising from each reference base. Dunn’s *post hoc* test indicated that significant differences were generally limited to pairwise comparisons of A vs. C, A vs. G, C vs. T, and G vs. T. Conversely, A vs. T and C vs. G pairings typically showed similar error profiles (fig S1.3). As before, correct call rates are omitted from the density plot, but are included in the statistical tests. Because significantly distinct error profiles were observed for the majority of contexts, we conclude that error rates are also generally base-dependent, and that a single error rate per library is too simplistic for use in the binomial test process.


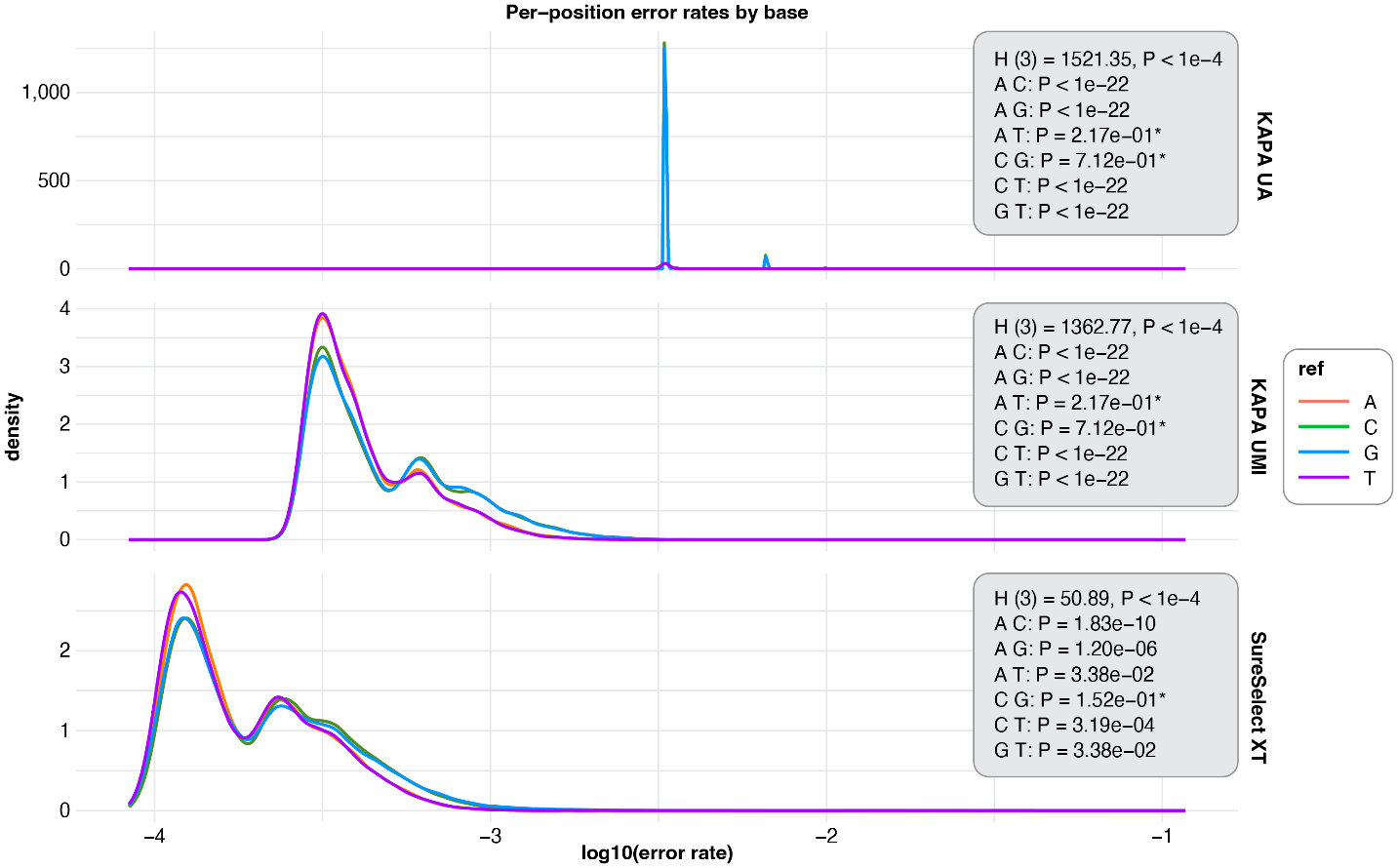


Figure S1.3; Error rate distributions for each reference base of the three library protocols are indicated by probability density curves. Curve color indicates reference base. Libraries are split by row. Kruskal-Wallis and Dunn’s test results are listed in grey legend boxes. Asterisks indicate non-significant differences in pairwise comparisons.

Finally, we compared *substitution-specific error rates per base*. Here, comparisons were made for each library, binning error rates for each reference base by specific substitution. Probability density curves (fig S1.4) showed fairly distinct patterns within each base, in that the most frequent substitution (that with the most right-skewed distribution) was uniquely base-specific and was associated with purine-purine or pyrimidine-pyrimidine substitutions. The minor substitution (highest leftmost peak, at low error frequency) in each base was either C (for A/G) or G (for C/T). We performed Kruskal-Wallis and Dunn’s *post hoc* tests for each base within each library and found significant differences in all pairwise comparisons across SureSelect XT and KAPA UMI libraries. The KAPA Universal Adapter library showed significant difference among approximately half of the pairwise comparisons in each base – though these results are likely impacted by the lower overall coverage for this sequencing run. Regardless, we conclude that, for at least the KAPA UMI and SureSelect XT libraries, that per-base error rates are also substitution-dependent.


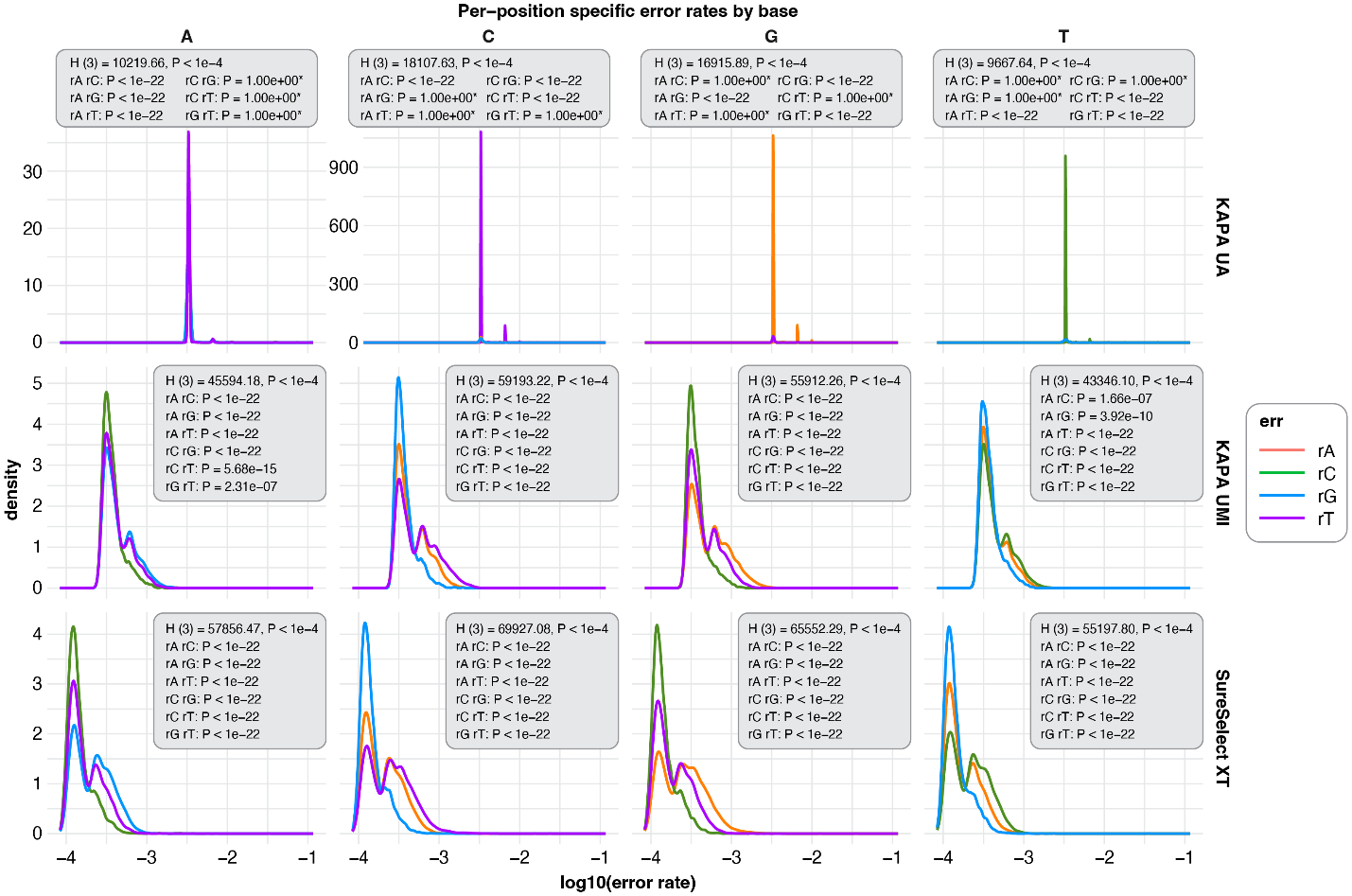


Figure S1. 4: Probability density curves indicate error rate distributions for specific mutations per reference base in each of the three library protocols. Reference bases per column, libraries per row. Curve color indicates specific substitution rate. Kruskal-Wallis and Dunn’s test results are listed in grey legend boxes. Asterisks indicate non-significant differences in pairwise comparisons.

This study presents data exclusively generated with SureSelect XT, and so specific error rates were determined based on error distributions for VG1 sequencing with this library preparation method. Supplemental figure 1.5 shows per-position distributions of specific substitution rates for each base. Vertical lines indicate the library mean per-position error rate in grey, the 95^th^ quantile value of per position error rates in black, and the specific 95^th^ quantile value for each distribution in its respective color.


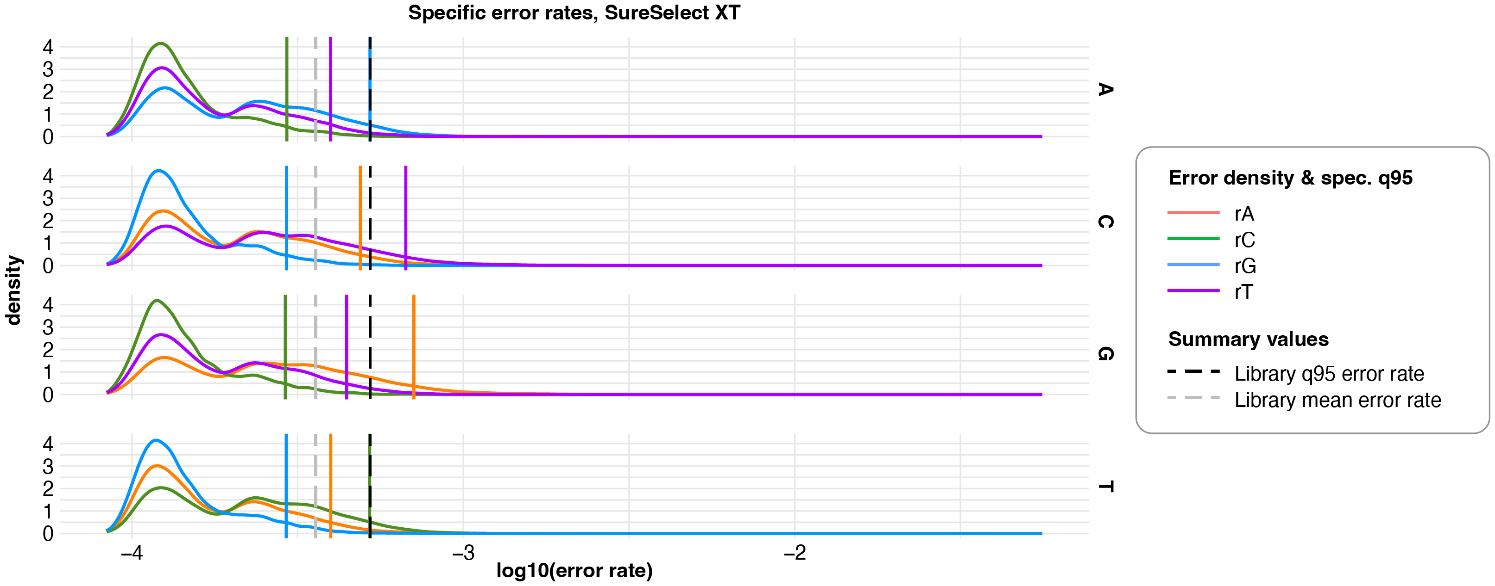


Figure S1. 5: Rescaled view of specific error rate distributions for the SureSelect XT library. Curve color indicates specific error rates per reference base. Colored vertical lines show 95^th^ quantile value for that base’s specific error distribution. Grey and black dashed vertical lines indicate the library mean and overall 95^th^ quantile error rates, respectively.

Conceptually, these vertical lines represent potential probabilities for use in the binomial test process. Our goal is to approximate the level at which an observed non-reference base frequency for a given position surpasses a reasonable noise threshold. The library mean rate would be too permissive of false positives for 8/12 potential substitutions. The library 95th quantile error rate would be too restrictive for the lower-frequency substitutions (e.g. C or G in all bases), and too permissive in the case of the most frequently observed T for reference C, and A for reference G. Thus, we opt to apply the 95^th^ quantile value for each specific substitution as our substitution probability for the binomial test process. This is thought to be the most reasonable and conservative value we could apply which minimizes false positives while being permissive enough to allow for naturally lower-frequency errors to still be detected. 95^th^ quantile substitution rates are shown in table S1.2.

| *Table S1.2 Observed 95^th^ quantile substitution rates* | | | | |
| --- | --- | --- | --- | --- |
|  | Ref = A | Ref = C | Ref = G | Ref = T |
| *Agilent SureSelect XT* | | | | |
| A | 0.99989074 | 0.000489 | 0.00070905 | 0.00039789 |
| C | 0.00029344 | 0.9998696 | 0.00029035 | 0.00052151 |
| G | 0.00052278 | 0.00029289 | 0.99987381 | 0.00029217 |
| T | 0.00039755 | 0.00067069 | 0.0004446 | 0.99988995 |
|  |  |  |  |  |
| *Roche KAPA Universal Adapter* | | | | |
| A | 1 | 0.00359712 | 0.00662252 | 0.00335571 |
| C | 0.00334897 | 1 | 0.00334448 | 0.00335571 |
| G | 0.00335571 | 0.00333333 | 1 | 0.00334448 |
| T | 0.00333333 | 0.00662252 | 0.00337212 | 1 |
|  |  |  |  |  |
| *Roche KAPA UMI* | | | | |
| A | 1 | 0.00103093 | 0.00142538 | 0.00087168 |
| C | 0.00069619 | 1 | 0.00069328 | 0.00098133 |
| G | 0.00100217 | 0.00069324 | 1 | 0.00068789 |
| T | 0.00088548 | 0.00143406 | 0.00100604 | 1 |

Non-reference base call counts are individually assessed at each genome position via one-tailed binomial test to determine whether the observed counts for that substitution are significantly greater than would be expected, based on empirical background 95th-quantile specific substitution rates. The raw binomial test results are imported into R (17, 18). The average base coverage was determined by calculating the sum of all nucleotides for each genome position (19-21). Nucleotide frequencies at each variant position were calculated by dividing the nucleotide counts by the sum. P-values (α) were corrected for multiple tests using the Bonferroni method with adjusted P-values (α’) = 0.05/k, where “k” denotes the number of binomial tests performed for each dataset. Non-significant substitutions are filtered from each dataset, and the resulting substitution signature is presumed to reflect the genotype of the non-dominant genome. Nucleotide frequencies at each variant position were visualized as a dot plot, excluding positions with a P-value > α’ and < 20X coverage (22, 23). The binomial test script additionally returns mean PHRED score *per base identity* at each position, which allows for manual assessment of spurious edge cases.

References

1. Bushnell. B. BBMap [BBTools is a suite of fast, multithreaded bioinformatics tools designed for analysis of DNA and RNA sequence data.]. Available from: sourceforge.net/projects/bbmap/.

2. Andrews S. FastQC: A Quality Control Tool for High Throughput Sequence Data [Online] 2010 [Available from: <http://www.bioinformatics.babraham.ac.uk/projects/fastqc/>.

3. Danecek P, Bonfield JK, Liddle J, Marshall J, Ohan V, Pollard MO, et al. Twelve years of SAMtools and BCFtools. Gigascience. 2021;10(2).

4. Institute B. Picard toolkit: Broad Institute; [Available from: <https://github.com/broadinstitute/picard>.

5. Bankevich A, Nurk S, Antipov D, Gurevich AA, Dvorkin M, Kulikov AS, et al. SPAdes: a new genome assembly algorithm and its applications to single-cell sequencing. J Comput Biol. 2012;19(5):455-77.

6. Li D, Liu CM, Luo R, Sadakane K, Lam TW. MEGAHIT: an ultra-fast single-node solution for large and complex metagenomics assembly via succinct de Bruijn graph. Bioinformatics. 2015;31(10):1674-6.

7. Boetzer M, Henkel CV, Jansen HJ, Butler D, Pirovano W. Scaffolding pre-assembled contigs using SSPACE. Bioinformatics. 2011;27(4):578-9.

8. Altschul SF, Gish W, Miller W, Myers EW, Lipman DJ. Basic local alignment search tool. J Mol Biol. 1990;215(3):403-10.

9. Bosi E, Donati B, Galardini M, Brunetti S, Sagot MF, Lio P, et al. MeDuSa: a multi-draft based scaffolder. Bioinformatics. 2015;31(15):2443-51.

10. Katoh K, Standley DM. MAFFT multiple sequence alignment software version 7: improvements in performance and usability. Mol Biol Evol. 2013;30(4):772-80.

11. Dotmatics. Geneious Prime 2023.1.1 [Available from: <https://www.geneious.com/>.

12. Smith T, Heger A, Sudbery I. UMI-tools: modeling sequencing errors in Unique Molecular Identifiers to improve quantification accuracy. Genome Res. 2017;27(3):491-9.

13. Schirmer M, D'Amore R, Ijaz UZ, Hall N, Quince C. Illumina error profiles: resolving fine-scale variation in metagenomic sequencing data. BMC Bioinformatics. 2016;17:125.

14. Quinlan AR. BEDTools: The Swiss-Army Tool for Genome Feature Analysis. Curr Protoc Bioinformatics. 2014;47:11 2 1-34.

15. Brander C, Suscovich T, Lee Y, Nguyen PT, O'Connor P, Seebach J, et al. Impaired CTL recognition of cells latently infected with Kaposi's sarcoma-associated herpes virus. J Immunol. 2000;165(4):2077-83.

16. Jones D, Ballestas ME, Kaye KM, Gulizia JM, Winters GL, Fletcher J, et al. Primary-effusion lymphoma and Kaposi's sarcoma in a cardiac-transplant recipient. N Engl J Med. 1998;339(7):444-9.

17. Team RC. R: A Language and Environment for Statistical Computing. 2023.

18. Team RS. RStudio: Integrated Development Environment for R. 2020.

19. Wickham H, Averick M, Bryan J, Chang W, McGowan LDa, François R, et al. Welcome to the tidyverse. Journal of Open Source Software. 2019;4(43):1686.

20. Wickham H, François R, Henry L, Müller K, Vaughan D. dplyr: A Grammar of Data Manipulation. 2023.

21. Wickham H, Hester J, Bryan J. readr: Read Rectangular Text Data. 2023.

22. Wickham H. ggplot2: Elegant Graphics for Data Analysis. 2016.

23. Wickham H, Seidel D. scales: Scale Functions for Visualization. 2022.
