# Supplementary material for: Sequencing of Kaposi’s Sarcoma Herpesvirus (KSHV) genomes from persons of diverse ethnicities and provenances with KSHV-associated diseases demonstrate multiple infections, novel polymorphisms, and low intra-host variance
Source: PLoS Pathog. 2024 Jul 15;20(7):e1012338. doi: 10.1371/journal.ppat.1012338 (PMC11271956; doi:10.1371/journal.ppat.1012338)
Supplement: S2 Document — (PDF) [file ppat.1012338.s011.pdf]

S3. Biochemical characterization of KSHVUNG-V9 variant

Highlights

1 We provide a detailed analysis of the glycosylase activity and binding affinity of KSHVUNG-V9 natural variant vs. WT KSHVUNG
2 KSHVUNG-V9 has lower affinity for dsDNA AP sites (the result of uracil excision)
3 KSHVUNG-V9 has elevated activity on uracil in dsDNA (U:G mismatch).

Materials and methods

Sequence analysis and structural predictions

149 KSHV ORF46 sequences from patient samples described herein were aligned to the GK18 reference sequence in Geneious Prime software. Homology modelling for the vUNG variants compared the crystal structure of the prototype V1 KSHV vUNG protein was performed in UCSF chimera.

Recombinant protein expression and purification

pet-DUET1-KSHVUNG or pet-DUET1-KSHVUNG-V9 was used for expression and purification of recombinant UNGs from BL21 *E. coli*. Expression and purification of recombinant KSHVUNG has been described before (Mu et al., 2023) KSHVUNG-V9 DNA sequence was synthesized (Twist Bioscience) and then amplified by PCR using primer pair (Duet1KSHVUNGEcoRIFP: ggatccgaattcgATGGACGCATGGTTGCAAC and Duet1KSHVUNGSalIRP: ggaagcgtcgacTTACTGCTCCAACAGGCCC) and were cloned into *EcoRI* and *SalI* restriction sites of the expression vector. Constructs were confirmed by sequencing.

E. coli BL21 cells (Invitrogen™ One Shot™ BL21 Star™ (DE3) containing pet-DUET1-KSHVUNG or V9 when OD600 reached 0.6. 12 L BL21 cells were harvested after overnight induction with IPTG and lysed by sonication in lysis buffer (20 mM Tris-HCl, 150 mM NaCl pH 7.5) and centrifuged at 12,000 g (Sorvall Lynx 6000 centrifuge, rotor F21–8X50Y) for 1 hr. 600 µl HisPur Cobalt resin (Thermo Cat. # 89964) was incubated with 600 ml cleared lysate, equally divided into 12× 50 ml conical tubes, rotated overnight in cold room with Fisherbrand™ Mini-Tube Rotators at speed 12 rpm. Cobalt resin was pelleted and resuspended with lysis buffer and loaded onto Poly-Prep Chromatography column from Bio-RAD (Cat. # 731–1550), which was equilibrated with 5 ml lysis buffer before addition of 600 µl HisPur Cobalt resin and washed with 2 ml 1% triton X-100 followed by 10 ml lysis buffer, then 2 ml 5 mM imidazole followed by 10 ml lysis buffer. His-tagged proteins were eluted with 300 µl 300 mM imidazole in lysis buffer and dialyzed against 20 mM Tris-HCl (pH 7.5), 50 mM NaCl, 1 mM dithiothreitol, 1 mM EDTA, and 10% glycerol. Proteins were stored at 50–100 µM in dialysis buffer at − 80 °C. Proteins were quantified after SDS-PAGE and Coomassie blue staining by band intensity quantification through comparison with BSA standard using Fiji software (Supplemental figure 6).

Fluorescence anisotropy assays

Binding of recombinant 6XHis-tagged UNG variants to dsDNA containing a single Tetrahydrofuran: G (THF:G) mismatch, a stable analog of an AP site, was measured by changes

in steady-state fluorescence depolarization (rotational anisotropy). fluorescein-labeled (FdT) ssDNA substrate containing a single THF was annealed with a complementary strand containing a G opposite the THF: 5′ AGAAAAGGGGAAAG(THF)AAAGAGGAAAGG(FdT)GAGGAGGT-3′. Reaction mixture (50 µl), containing fluorescein-labeled DNA (10 nM) in buffer (50 mM potassium acetate, 20 mM Tris-acetate (pH 7.9), 10 mM magnesium acetate, 1 mM DTT), was incubated with increasing concentrations (0–10000 nM) of UNG at room temperature. Rotational anisotropy was measured using a BioTek™ Synergy™ H4 Hybrid Microplate Reader. Samples were excited with vertically polarized light at 485 nm, and both vertical and horizontal emission were monitored at 528 nm (20 nm band pass). The microscopic dissociation constant (Kd) is defined as the concentration of UNG at which half of the total THF:G dsDNA is bound. Apparent dissociation constants (Kd) were obtained by fitting the data to a rectangular hyperbolic curve for noncooperative binding using Graphpad Prism 9.0 software. The data were calculated from three independent experiments.

UDGase assay to measure UNG-specific activity on U in ssDNA or G:U mismatch in dsDNA. UNG enzymatic activity assays were previously described (Mu et al., 2023). KSHV UNG and KSHV UNG-V9 enzymatic activities were measured using Fluorescein-labeled 36-nt ssDNA 5′AGAAAAGGGGAAAGUAAAGAGGAAAGG(FdT)GAGGAGGT-3′ or the same Uracil-containing oligo annealed to complementary strand with a G opposite U for dsDNA. UDGase reactions (30-µl volume) were carried out in a buffer containing 50 mM potassium acetate, 20 mM Tris-acetate (pH 7.9), 10 mM magnesium acetate, 1 mM DTT, 333 nM of the substrate DNA. Following incubation with UNG at 37 °C, the reactions were quenched by a double extraction with phenol/chloroform/isoamyl alcohol (25:24:1). 50 nM NaOH was added to extracted DNA and heated for 5 min at 95 °C to accomplish cleavage of AP site. UDGase reactions after NaOH treatment were mixed with equal volume of stop solution (9.5 ml formamide, 0.4 ml 0.5 M EDTA pH 8.0 and 0.1 ml H2O) and resolved with 8 M Urea containing denaturing 16% PAGE in 1XTBE buffer. A Typhoon scanner (Amersham Typhoon 5) biomolecular imager was used to image gels and determine DNA band intensity. Quantification was via Image J (version 1.53c) and specific activity was calculated as nanomoles of uracil removed from DNA substrate/min/µg of enzyme. This was determined in the linear range of protein concentration and incubation times. Summary of activity was determined from three independent experiments and error bars were calculated based on standard error of mean. Statistical analysis was via unpaired student T-test, and dissociation constant was analyzed by Extra sum of squares F-test.
